# LiG Metrology, Correlated Error, and the Integrity of the Global Surface Air-Temperature Record

**DOI:** 10.3390/s23135976

**Published:** 2023-06-27

**Authors:** Patrick Frank

**Affiliations:** Scientific Staff Emeritus, SLAC National Accelerator Laboratory, Stanford University, Menlo Park, CA 94025, USA; pfrank@slac.stanford.edu

**Keywords:** systematic measurement error, metrology, resolution, uncertainty, thermometer glass, Joule-drift, LiG non-linearity, correlated error, SST, global air temperature

## Abstract

The published 95% uncertainty of the global surface air-temperature anomaly (GSATA) record through 1980 is impossibly less than the 2σ = ±0.25 °C lower limit of laboratory resolution of 1 °C/division liquid-in-glass (LiG) thermometers. The ~0.7 °C/century Joule-drift of lead- and soft-glass thermometer bulbs renders unreliable the entire historical air-temperature record through the 19th century. A circa 1900 Baudin meteorological spirit thermometer bulb exhibited intense Pb X-ray emission lines (10.55, 12.66, and 14.76 keV). Uncorrected LiG thermometer non-linearity leaves 1σ = ±0.27 °C uncertainty in land-surface air temperatures prior to 1981. The 2σ = ±0.43 °C from LiG resolution and non-linearity obscures most of the 20th century GSATA trend. Systematic sensor-measurement errors are highly pair-wise correlated, possibly across hundreds of km. Non-normal distributions of bucket and engine-intake difference SSTs disconfirm the assumption of random measurement error. Semivariogram analysis of ship SST measurements yields half the error *difference* mean, ±½Δε_1,2_, not the error mean. Transfer-function adjustment following a change of land station air-temperature sensor eliminates measurement independence and forward-propagates the antecedent uncertainty. LiG resolution limits, non-linearity, and sensor field calibrations yield GSATA mean ±2σ RMS uncertainties of, 1900–1945, ±1.7 °C; 1946–1980, ±2.1 °C; 1981–2004, ±2.0 °C; and 2005–2010, ±1.6 °C. Finally, the 20th century (1900–1999) GSATA, 0.74 ± 1.94 °C, does not convey any information about rate or magnitude of temperature change.

## 1. Introduction

The first constructions of hemispheric or global air-temperature anomaly trends, though admirable, did not consider instrumental reliability [1,2,3]. Meteorological air temperatures were accepted at face value. Mitchell’s 1953 assessment of artefacts entering meteorological air temperatures focused on instrumental site relocations and the urban heat island effect (“*city influences*”) [4]. Interestingly, Mitchell also mentioned the spurious secular trends produced by aging thermometers, which have since been ignored. Nevertheless, he did not discuss the systematic measurement errors arising from solar irradiance or inadequate wind speed [5,6]. Temperature records contaminated by systematic error can pass all the standard statistical and comparative tests used to establish meteorological reliability [7].

Recognition that uncontrolled environmental variables have an impact on the accuracy of meteorological liquid-in-glass (LiG; a list of acronyms follows the Acknowledgements Section) thermometers housed within the naturally ventilated louvered Stevenson screen or the equivalent cotton region shelter (CRS) was already wide-spread in the 19th century. In 1879, Frederic Gaster reported on Griffiths’ 1869 Stevenson screen calibration experiments at the Strathfield-Turgiss rectory [8]. Gaster noted that, “*We do not know even now how far from truth the readings recorded are on any stand, but only how far they differ from a certain standard, and that standard is believed to be somewhat faulty*” [9]. Nevertheless, 150 years later, the mistaken notion still persists [10] that the naturally ventilated louvered Stevenson or CRS shield solved the problems of uncontrolled environmental variables and loss of accuracy in meteorological air-temperature measurements. Meteorological air temperatures continue to be accepted at face value [11,12,13].

Trending global air temperature has been of increasing climatological concern for more than 40 years [14,15,16,17,18]. Since 1988, climatology has focused heavily on global air temperature [19,20,21,22,23,24]. Consequently, the reliability of the global air-temperature record is of central importance. Remarkably, however, over this time there has been no assessment of the reliability of the liquid-in-glass (LiG) thermometer as a meteorological instrument.

Instrumental calibration is basic to accurate measurement [25,26,27,28,29,30]. Field calibrations of land-surface air-temperature sensors deployed in the United States Historical Climatology Network (USHCN) have invariably revealed systematic temperature measurement errors deriving from uncontrolled environmental variables [8,31,32,33,34,35,36,37,38,39,40]. These environmental variables include direct solar irradiance or surface albedo reflectance, which cause heating within naturally ventilated louvered thermometer screens, or low wind speed (<5 m/s) that is insufficient to ventilate the sensor with external ambient air [40,41,42,43,44,45]. Systematic errors under field conditions vary unknowably across sign and magnitude, and have a negative impact on measurement accuracy [30,46,47,48].

Many studies reporting hemispheric or global air-temperature averages do not discuss sensor-measurement error at all [17,19,49,50,51,52,53,54,55,56,57,58]. When it is mentioned, air-temperature-measurement error is invariably described as random [10,59,60,61,62,63,64]. Quayle and associates mentioned systematic error due to shelter heating [65]. However, reconciliation of this source of error has not found its way into the air-temperature record. Rather, the accounting of systematic error has been limited to, “*nonclimatic sources …* [*t*]*hermometer exposure change bias …* [*u*]*rban biases … due the local warming effect* [*and*] *incomplete spatial and temporal coverage*” [11]. The systematic measurement errors from solar irradiance, or surface reflection, or insufficient ventilation of the instrument were neither mentioned nor considered.

Daily systematic measurement errors due to environmental variables put a significant and permanent uncertainty of unknown sign or magnitude into a station monthly mean air temperature. This uncertainty can be estimated only by way of careful field-calibration experiments [7,31,32,35,36,40,66,67,68]. Measurement uncertainties revealed by field-calibration experiments propagate into and condition an air-temperature mean [7,66,69].

The same general assumption of random sensor-measurement error attends contemporaneous discussions of bucket and ship engine-intake sea-surface temperature (SST) measurements [70,71,72,73,74,75,76]. Most modern workers account systematic error as stemming only from methodological imprecision, which is treated as a single-valued offset bias that can be removed by differencing [73,77,78]. However, SST field-calibration experiments led earlier researchers to be more critical and far less sanguine [79,80,81,82]. Thus, the 1975 meteorological data set compiled by the National Center for Atmospheric Research (NCAR) noted that, “*the RMS of differences between ship observations and Navy analyses based mostly on ship observations is [±]1.4 °C*” (RMS is root-mean-square) [83]. Likewise, Weare, and Gleiker and Weare, surveyed sensor-measurement error and concluded that ±0.5 °C systematic uncertainty conditioned both global land-surface and sea-surface temperatures [84,85]. However, these cautions have not found their way into the published record.

Pairwise field SST comparisons at 5 km separation (immersive co-location) revealed a buoy–buoy RMS difference (*N = 6890*) of ±0.15 °C, while the ship–buoy (engine-intake) RMS difference (*N = 840*) was about ±0.9 °C [86,87]. There was no guarantee that such differences reflect random error. Likewise, when the temperature sensor in drifter buoys were field-calibrated against ship-deployed conductivity–temperature–depth (CTD) sensors, SST measurement uncertainties of ±0.14 or ±0.28 °C were revealed [88]. Similar CTD field calibrations of ARGO buoys along a 36° N Atlantic transect revealed an RMS error of ±0.6 °C [89]. These recent RMS uncertainties in measured SSTs are similar in magnitude to the earlier field calibrations of SST measurements that produced cautionary judgments [79,80,81]. Systematic temperature-sensor measurement errors that arise from uncontrolled environmental variables are invariably larger than 0.1 °C and can display uncertainty distributions that are far from normal and, typically, biased warm [31,36,80,81,90,91].

The impact of systematic land- and sea-surface temperature-sensor measurement errors on the reliability of the global air-temperature record has more recently been estimated [7,66,92]. In this work, the instrumental reliability of the global air-temperature record is comprehensively assessed. Detection and resolution limits, LiG non-linearity, and Joule-drift in LiG thermometers are quantitatively examined. The relevant compositional history of thermometer glass is included to provide context, augmented with X-ray fluorescence examination of a very early 20th century meteorological thermometer bulb. Previously unknown behavior of air-temperature-sensor field-calibration measurement error is described. This is followed by a general test of the universal assumption of strictly random sensor error in the land-surface and sea-surface temperature measurements. The semivariogram and transfer-function methodologies are critically examined. Finally, the lower-limit resolution of meteorological LiG thermometers is combined with the systematic measurement errors within both land-surface and sea-surface temperatures to produce a new lower-limit estimate of uncertainty in the global averaged surface air-temperature anomaly record.

## 2. Facilities and Methods

Lead (Pb) X-ray fluorescence (XRF) was measured on the bulb glass of a 1900-vintage Baudin alcohol-filled liquid-in-glass (LiG) meteorological thermometer (Baudin no. 15774), brought into use by the U.S. Weather Bureau. In 1960, the thermometer was donated to the National Museum of American History (NMAH), Washington, D.C. (item PH.317453). The meta data provided by the NMAH are as follows: “*Alcohol-in-glass thermometer with a long cylindrical bulb. The milk white back of the tube is marked “Thermomètre Baudin No. 15774 gradué d’apres l’Échelle Normale Internationale* (*1902.9*). (*Thermomètre Baudin No. 15774 graduated from the Échelle Normale Internationale* (*1902.9*))*” The scale on the front of the tube extends from −70.0 to +30.0 degrees, graduated in fifths. There is a safety bulge at the top of the tube*”.

The overall dimensions are 20^1^/_4_ in × ^1^/_4_ in (51.435 cm × 0.635 cm) with a −70 to +30 °C scale, scored with 0.2 °C divisions. The factory calibration was evidently carried out during 1902 September. Measurement accuracy is also about ±0.2 °C, which is typical of LiG meteorological thermometers, even those with 0.1 °C graduations [93].

The XRF spectroscopic measurement was carried out by Dr. Kristen Frederick-Frost, NMAH Division of Medicine and Science Curator at the National Numismatic Collection, NMAH, using a Bruker S1 TITAN/TRACER 5i hand-held X-ray spectrometer. X-ray fluorescence was collected for about 15 s. The spectrometer output was reformatted into two-column ASCII by Mr. Artur Neves, Department of Conservation and Restoration, NOVA School of Science and Technology, Portugal.

Joule-drift was examined using the record of James Joule’s 1844 Dancer-manufactured mercury LiG Fahrenheit thermometer, which was scored to 13 graduations per °F [94,95]. The Dancer thermometer readings were converted to Celsius as (1/13) × (5/9) and dates of ice-point calibration were assumed to be mid-month.

All numerical or graphical analysis was carried out using the Kaleidagraph analytics package (Synergy Software). Normality of an error data set was tested using the Shapiro–Wilk (S–W) test [96,97,98]. Data points *N ≥ 50* were required to accept a S–W normality test as fully valid [97]. Data points from published graphics were digitized using DigitizeIt software (I. Bormann).

## 3. Results

### 3.1. LiG Thermometers: Resolution, Linearity, and Joule-Drift

#### 3.1.1. Resolution

Until relatively recently, the U.S. National Institute of Standards and Technology (NIST) carried out detailed calibrations and evaluations of LiG thermometers [99,100]. NIST publications list the visually indistinct physical imperfections that can degrade the accuracy of a LiG thermometer, including “*changes in bulb volume, microscopic alterations in glass geometry at elevated temperature, microscopic cracking, degradation of the thermometer liquid*”, and endogenous solids, such as glass particles in the capillary [101,102]. Microscopic inspection to detect such flaws preceded calibration of LiG thermometers at NIST [102]. The presence and influence of physical imperfections that may have affected LiG thermometers over the historical air-temperature-measurement record are unknown.

After about year 2000, NIST calibration and testing of LiG thermometers utilized a computer-driven digital camera and 10× magnification to visualize the meniscus of the liquid column [100,103]. System resolution was 1/34 of the smallest stem division, equivalent to ±0.03 °C for a 1 °C/division LiG thermometer. Under this high-precision visualization, calibration will yield the physical resolution limit of the thermometer. That is, the visualization errors are negligible, leaving calibration errors stemming only from the imperfections and the sensitivity of the thermometer itself.

NIST calibration of two 1 °C/division full-immersion mercury LiG thermometers, with four repetitive readings taken every 25 °C between 0–100 °C, yielded a 2σ = ±0.11 °C (95% CI) resolution limit [100]. The same ultimate 2σ = ±0.12 °C uncertainty was found in an interlaboratory comparative calibration of three 0.1 °C/division full-immersion Hg LiG Thermo-Schneider thermometers [104]. The ±0.12 °C uncertainty emerged despite the fact that the visualization apparatus provided 1/5 (±0.02 °C) division resolution. This 2σ = ±0.11 °C represents the resolution (detection) limit—the lowest limit of uncertainty—that can be associated with a temperature measured using a meteorological surface-station mercury LiG Celsius thermometer. A meteorological LiG air-temperature thermometer may be considered total immersion, as the entire instrument is bathed in ambient air.

Prior to year 2000, LiG thermometer calibrations at NIST employed visualization by eye, which is the standard method used to obtain LiG thermometer readings at meteorological stations. The uncertainty attending a LiG thermometer reading taken by eye (visual repeatability) is 1/4 of a scale division [101,105]. For a calibrated 1 °C/division mercury or alcohol (spirit) meteorological LiG thermometer, the true air temperature is taken to be somewhere within ±0.125 °C of the measurement as read by eye. The reported uncertainty associated with this rectangular probability distribution is 1σ = (±0.125/$\sqrt{3}$) °C = ±0.072 °C. The lower-limit estimate of laboratory accuracy for a visually-read 1 °C/division full-immersion mercury LiG meteorological thermometer combines in quadrature the intrinsic resolution limit plus the repeatability. Thus, 2σ = 1.96 × $\sqrt{{\left(0.055\right)}^{2}+{\left(0.072\right)}^{2}}$ = ±0.178 °C. This is the minimum confidence interval that must condition any meteorological air temperature, or a mean of air temperatures. The ±0.18 °C, 95% lower limit of uncertainty, is the laboratory ideal instrumental-accuracy limit, representing resolution combined with visual repeatability. If a LiG thermometer suffers ice-point calibration drift and is recalibrated, the uncertainty from visual repeatability again enters. For a 1 °C/division LiG thermometer, the new lower limit of uncertainty following recalibration is then 2σ = 1.96 × $\sqrt{{\left(0.055\right)}^{2}+{\left(0.072\right)}^{2}+{\left(0.072\right)}^{2}}$ = ±0.23 °C [101].

In constructing an air-temperature anomaly, both every station mean and the reference normal will each carry, at least, the lower limit ±0.18 °C as the 95% RMS uncertainty. Taking the (mean minus normal) difference to obtain an annual air-temperature anomaly requires combining the respective uncertainties in quadrature [106]. This calculation yields $2\sigma =1.96\times \sqrt{{\left(0.0.0908\right)}^{2}+{\left(0.0.0908\right)}^{2}}$ = ±0.25 °C as the 95% lowest-limit uncertainty bound in any LiG-derived mean air-temperature anomaly.

However, NBS/NIST calibration circulars published between 1911–1994 tabulated the accuracy for calibrated full-immersion mercury LiG thermometers of 1 °C/division to be ±0.1–0.2 °C following correction for all known systematic errors [102,107,108,109]. The tolerance limit—the maximum of error in an uncalibrated thermometer—was ±0.5 °C. Accepting the 1911–1994 NBS/NIST intermediate ±0.15 °C accuracy value for good-quality full-immersion mercury LiG thermometers over the 20th century, along with the standard ±0.072 °C visual repeatability, the lower-limit 95% uncertainty becomes 2σ = 1.96 × $\sqrt{{\left(0.15\right)}^{2}+{\left(0.072\right)}^{2}}$ = ±0.326 °C in both any pre-2000 meteorological air temperature and in the RMS uncertainty of a 30-year air-temperature reference normal. In this case, the 95% lower limit of experimental uncertainty in a pre-2000 mean air-temperature anomaly becomes 2σ = $1.96\times \sqrt{{\left(0.166\right)}^{2}+{\left(0.166\right)}^{2}}$ = ±0.46 °C.

This NIST calibration range of accuracy marks the lower limit of uncertainty for temperatures read from full-immersion mercury LiG meteorological thermometers scaled to 1 °C/division, prior to year 2000. As noted above, NIST automated the visualization for their calibration procedure after 2000 [103], which improved the accuracy of calibration. This greater level of LiG calibration accuracy yields an uncertainty of 2σ = ±0.25 °C in a post-2000 annual LiG temperature anomaly, as noted above. In constructing a global air-temperature anomaly, this uncertainty enters as the weighted fraction of LiG thermometer temperatures. Table 1 summarizes the base-level uncertainties conditioning the Hg LiG air temperatures.

The empirical uncertainty of alcohol-filled LiG low-temperature thermometers is about twice that of the Hg LiG instruments [99,102]. The estimated pre-year-2000 ± 2σ uncertainty in an alcohol LiG Celsius thermometer, per measurement, is then ±0.62 °C or is ±0.87 in an anomaly. Post year 2000, the uncertainties are ±0.23 °C per measurement and ±0.32 °C in an anomaly. The same absolute values of resolution and repeatability are applicable to Fahrenheit thermometers, with uncertainties improving by 5/9 over the Celsius LiG instrument. Table 1 lists laboratory resolution limits only. Uncertainties following from non-linearity and Joule-drift (see below) are not included.

#### 3.1.2. Linearity

Degradations of LiG thermometer resolution due to physical deterioration are episodic and local. However, non-linearity of response is a small but universal source of measurement error in LiG thermometers [93,110]. Non-linearity of LiG thermometers arises because the mercury or ethyl alcohol in the capillary does not expand uniformly with temperature [111,112]. Following two-point calibration at 0 °C and 100 °C, a meteorological thermometer will parabolically depart from the correct temperature above 0 °C and recover at 100 °C, reaching a maximum of error near 50 °C [110,112,113].

**Figure 1:**
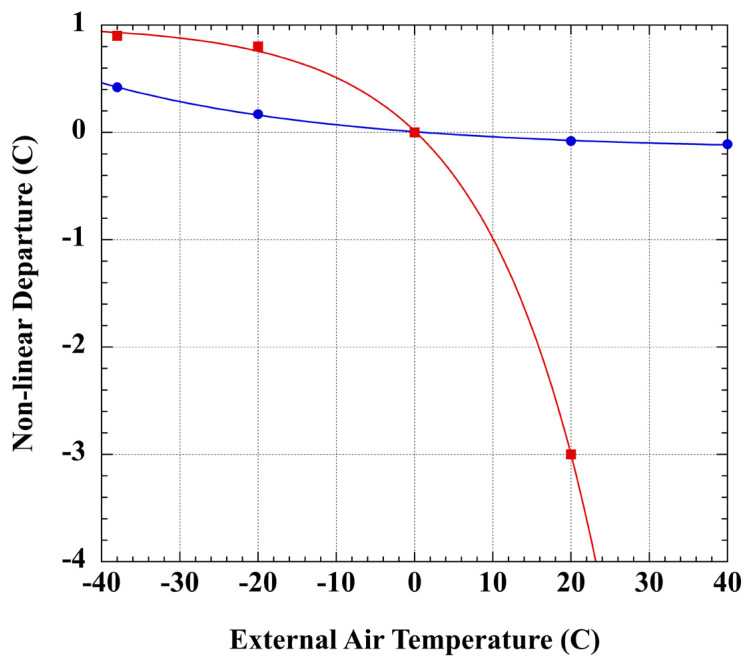
Non-linear departures of measured air temperature within LiG thermometers calibrated at 0 °C. (blue circles), mercury-filled thermometer; and (red squares), ethanol-filled (spirit) thermometer [112]. The lines are exponential fits to the points. Mercury: *y = [0.168 × exp(—0.033x)] — 0.161, r^2^ = 0.9991*; ethanol: *y = [—0.983 × exp(0.070x)] + 0.998, r^2^ = 0.9997*.

Figure 1 shows these errors over the range of meteorological temperatures. For mercury thermometers, the error is relatively small, however, error can be significant in spirit thermometers. The World Meteorological Organization does not include non-linearity among the errors specific to spirit thermometers [114]. Assuming alcohol LiG thermometers dominate the historical daily minimum temperature record, and mercury LiG instruments the daily temperature maxima, then from Figure 1 any uncorrected minimum temperatures in Winter will have been recorded as too warm and in Summers as slightly too cool. For example, an uncorrected single Winter day of −10.00 °C minimum (alcohol) and 10.00 °C maximum (Hg) temperatures, corrects to −9.49 °C and 9.96 °C, respectively. The nominal 0 °C average thus corrects to 0.24 °C.

Although the correction is modest, the error due to non-linearity of response is present in the historical LiG air-temperature measurements entering the global average. Correction of this error is not mentioned in published work. The larger relative magnitude of the alcohol correction implies a slightly warmer past than presently recognized, which should be considered in estimates of climate warming. Correction of past temperatures for non-linearity of response requires knowing the characteristics of the land-surface-station and ship-borne LiG thermometers.

#### 3.1.3. Joule-Drift

The slow upward drift in ice-point calibration temperature due to contraction of the glass bulb of a LiG thermometer was first reported in 1808 [112]. A detailed investigation of this problem was reported in 1837. Bulb contraction in LiG thermometers occurs because residual strain remains in the glass after manufacture. As this strain is slowly released the bulb contracts, which, in turn, causes the indicating liquid to rise in the stem. An artifactual increase in measured temperatures is thereby produced [112,115,116]. Mid-19th century thermometer recalibrations after decades to centuries of use revealed ice-point calibration shifts of 0.3–0.6 °C due to bulb contraction [112,115,117]. Left uncorrected, bulb contraction in early meteorological LiG thermometers will have produced a false warming trend extending across decades.

The changes in volume of LiG thermometer bulbs came under detailed examination in the mid-19th century and continued well into the 20th [94,117,118,119,120,121,122,123,124,125,126,127]. Starting in April 1844, James Joule began an ice-point drift experiment using a long high-resolution Dancer Hg-LiG Fahrenheit thermometer that had been manufactured a few months earlier. Joule performed 13 ice-point temperature calibrations on this thermometer through to December 1882 [95,128]. By that time, it was common knowledge that ice-point drift was inevitable and that the relaxation behavior of each thermometer was unique [117,125,129]. Following Joule’s death in 1889, three more ice-point calibrations were obtained on the same Dancer thermometer during 1892–1894 [130]. The final calibration was performed in 1930, by which time the departure was 0.67 °C [131,132]. Unfortunately, the glass composition of Joule’s Dancer thermometer is unknown and the thermometer itself was lost in 1942 during a WWII air-raid.

Sydney Young first reported that the rise in ice-point temperature in Joule’s LiG thermometer was exponential with time [94]. Young’s finding was later grounded in physical theory [121]. The upward drift in LiG thermometer ice-point temperatures was found to follow one or more relaxation processes, *T* = *a*(1 − *e*^−*kt*^), where *T* is temperature, *a* is a constant, *k* is a rate constant, and *t* is time in years [118].

**Figure 2:**
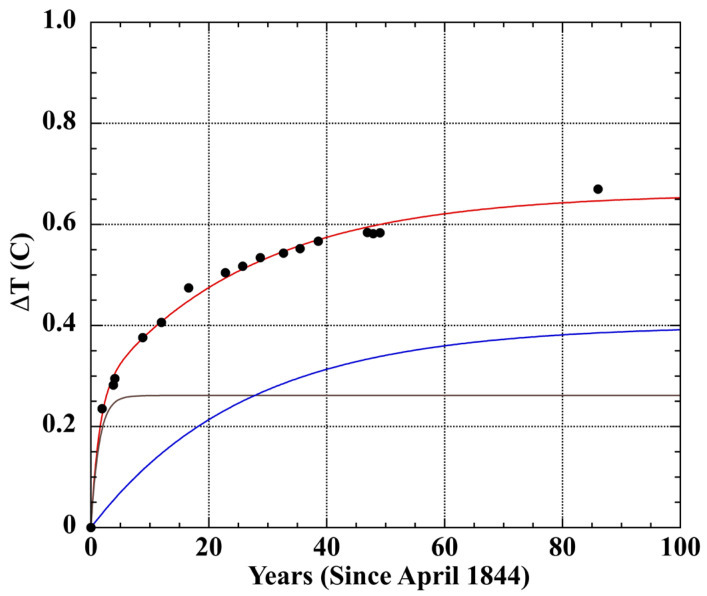
Ice-point creep of James Joule’s Dancer-manufactured 19th century liquid-in-glass (LiG) mercury thermometer. (Points), variation in ice-point calibration temperature from April 1844 [128,130,131]. (Red line), double Taylor-Noyes exponential fit to the points, *r^2^ = 0.994* (see text). (brown line), exponential 1: *0.26 ± 0.02 × (1 — exp(—0.73 ± 0.17 × year)); t_1/2_ = 1.0 ± 0.2 year*. (blue line), exponential 2: *0.40 ± 0.02 × (1 — exp(—0.038 ± 0.005 × year)); t_1/2_ = 18 ± 2 year*.

Figure 2 shows the 86 years of measurement creep in Joule’s Dancer thermometer. The points were fit with two Taylor–Noyes exponentials [118]. These imply at least two independent relaxation mechanisms within the bulb-glass, with half-lives of 1 ± 0.2 year and 18 ± 2 years. Thus, about 180 years (10 half-lives) are required for the secular change in the Dancer bulb volume to become negligible. The more rapid process diminishes to about 3% of its initial rate after 5 years. This explains the confidence among 19th century manufacturers and researchers in the accuracy of thermometers that had been calibrated a few years after filling. However, Figure 2 indicates this confidence was misplaced. Joule’s ice-point measurements have been investigated extensively [133,134,135,136]. However, the two exponential phases shown in Figure 2 were not resolved.

**Figure 3:**
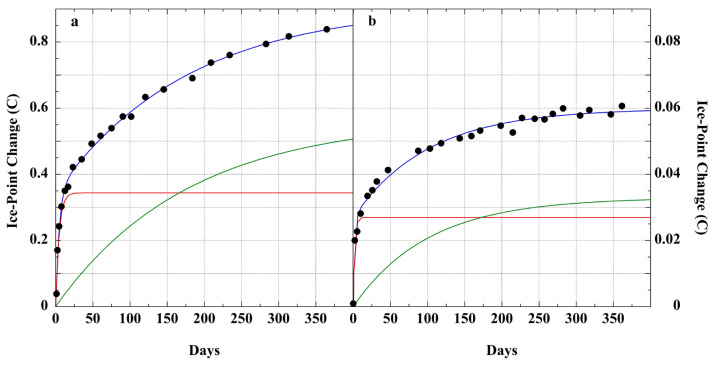
(points), mercury LiG thermometer ice-point drift measured during roasting at 262 ± 1 °C [122]. The blue fitted line is the sum of two Taylor–Noyes exponentials [*a × (1 − e^−kt^)*] [118]. (**a**) Corning 0041 potash–soda–lead–silica glass [152]; (red), *a_1_ = 0.34 ± 0.01, k_1_ = 0.22 ± 0.02 d^−1^, t^1/2^ = 3 d*; (green), *a_2_ = 0.57 ± 0.02, k_2_ = 0.0056 ± 0.0007 d^−1^, t^1/2^ = 119 d, r^2^ = 0.996*. (**b**) Corning 1720 borosilicate glass [153]; (red), *a_1_ = 0.027 ± 0.001, k_1_ = 0.48 ± 0.09 d^−1^, t^1/2^ = 1.4 d*; (green), *a_2_ = 0.033 ± 0.001, k_2_ = 0.010 ± 0.001 d^−1^, t^1/2^ = 70 d; r^2^ = 0.989*.

Prior to 1885, thermometers were manufactured from glasses typified by “Thuringian” glass (Table 2), or from lead (PbO, litharge) glass, each of which contained significant fractions of both sodium and potassium ions. Nineteenth-century lead-glass thermometers exhibited an increased rate of bulb contraction [125,137,138,139,140]. Following the discovery of bulb-contraction, the most careful manufactories scored and calibrated their LiG thermometers several months after being filled and sealed [112]. After about 1880, Kew Observatory manufacture of LiG thermometers included heating in an oil bath for 2–3 weeks prior to calibration, and at a temperature exceeding the limit of measurement [141]. However, these were only partial solutions, because, even after treatment at elevated temperature, ice-point drift could continue for decades at ambient temperature (*cf.* Figure 3).

In 1884 Otto Schott and Ernst Abbe traced bulb contraction to the mixed alkali effect (MAE), i.e., glass compositions that included significant fractions of both potassium and sodium oxides [123,124,142,143,144,145,146,147]. Their Jena hard glass 16^III^ and 59^III^, manufactured after 1885, introduced B_2_O_3_ into thermometer glass. The new process simultaneously excluded lead oxide and restricted alkali metal usage to Na_2_O. These compositional adjustments reduced bulb contraction and thermometer ice-point drift by about 10-fold. The molecular mechanism of bulb stress relief includes movement and polymerization of silicate ions [148]. The mechanistic dynamics are complex and remain an open area of research [142,145,148,149,150,151].

Figure 3 illustrates the dramatic difference of lead-glass (Corning 0041) versus hard borosilicate glass (Corning 1720) in thermometer-bulb contraction. Each exhibits a faster and slower process. After 360 days at 262 °C, the lead-glass thermometer exhibited a ~14-fold greater shift in ice-point. Table 2 presents representative glass compositions of LiG thermometers exhibiting the different behaviors.

Both Joule’s Dancer thermometer (Figure 2) and the two test thermometers (Figure 3) indicate similar simultaneous fast and slow relaxation processes. The modern lead–silica LiG thermometer produced the expected larger and more extensive ice-point drift. The improvement realized in a borosilicate glass LiG thermometer is evident in the 14-fold reduction in ice-point drift at 360 days.

The glass composition of Joule’s Dancer thermometer is unknown. The notion that it was the borosilicate of Jena glass 59^III^ is not tenable [134,136]. Jena borosilicate glasses were not developed until 1885 [123,143], 41 years after Joule acquired his thermometer. Although William Harcourt experimented with borosilicate glass in the mid-19th century, his focus was on optical instruments [123,154]. B_2_O_3_ was not used in thermometer glass until after the experiments of Schott and Abbe at the Jena glassworks in 1883–1884 [123,143]. The new Jena 59^III^ borosilicate glass produced thermometers with a greatly diminished ice-point drift, which is inconsistent with the 0.67 °C drift exhibited by Joule’s Dancer thermometer. The Joule thermometer drift is consistent with lead–silica glass (compare Figure 2 with Figure 3a vs. Figure 3b), which was in general use for thermometers in the mid-19th century. For example, a Scottish scientific thermometer dating from the first decades of the 19th century was composed of glass containing 20.2% PbO [140].

**Table 2:** Relevant Thermometer Glass Compositions.

Glass Type	SiO_2_	Na_2_O	K_2_O	CaO	B_2_O_3_	Al_2_O_3_	PbO	Reference
Silica-lead ^a^	68	10	6	1	---	---	15	[152]
Borosilicate ^a^	80	14	---	---	14	2	---	[152]
Corning 0041	50.1	6.6	1.5	---	---	1.9	39.9	[155]
Corning 1720 ^b^	62	1	---	8	5	17	---	[153]
Jena 59^III c^	72	11	---	---	12	5	---	[156]
Thuringian ^d^	68.7	15.9	7.3	5.7	---	2.1 ^e^	---	[144]
Kew ^f^	53.9	1.7	8.5	0.56	---	0.48	34.5	[157]
Kew ^g^	53	0.5	11.5	---	---	0.5 ^d^	34	[158]

^a^ Representative compositions. ^b^ Includes ~7% MgO [159]. ^c^ Single alkali reduces thermometer bulb contraction. ^d^ Typical of German and French common thermometer glass prior to 1885; included ~0.24% MgO. ^e^ May also include Fe_2_O_3_. ^f^ From the early 1850′s; mean of analyzed percentages in ref. [160]; included traces of Fe_2_O_3_ and MnO. ^g^ Introduced around 1880 [139].

### 3.2. Lead Glass

In the early 1850s, the Kew observatory purchased a lead glass (Choisy le Roi crystal [161]) thermometer from Paris chemist and instrument-maker Henri Regnault for use as a standard applied to their own manufacture of thermometers [157]. From this mid-19th century start, Kew Observatory came to supply high-quality standard thermometers composed, bulb and stem, of glass with ~34% lead oxide (Table 2) to European and American experimenters [139,158]. E. H. Griffiths mentioned that soft-glass thermometers were in wide English use as late as 1894 [162]. During the late 19th century, both lead glass and hard glass were used in manufacture of thermometers by Tonnelot and his successor Baudin in Paris [117,162,163]. However, by 1903 Baudin moved to lead-free French hard glass (*verre dur*) [110,164].

**Figure 4:**
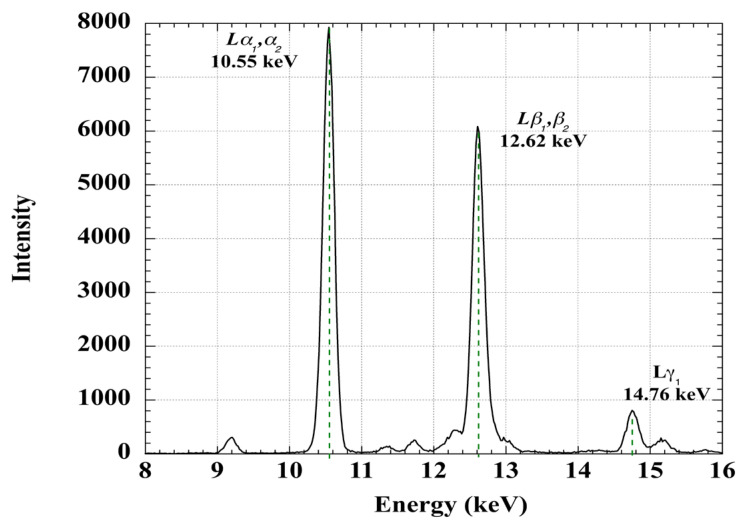
Lead (Pb) L-edge X-ray fluorescence spectrum of the glass bulb of Baudin no. 15774 liquid-in-glass (LiG) alcohol-filled thermometer (−70 to +30 °C). National Museum of American History item ID PH.317453 (see Facilities and Materials for details).

Figure 4 shows the Pb L-edge X-ray fluorescence (XRF) spectrum of the bulb glass of a Baudin meteorological LiG spirit thermometer, manufactured around 1900 and purchased by the U.S. Weather Bureau (see Facilities and Methods).

The noise intensity of the Pb L-edge XRF spectrum is within the width of the line, indicating considerable lead. This thermometer was purchased by the U.S. Weather Bureau in 1902, and donated to the National Museum of American History in 1960. Thus, lead-glass meteorological LiG thermometers continued to be manufactured and brought into service as late as 1900.

### 3.3. Thermometer Field Calibration and Measurement Error

Field-calibration experiments of naturally ventilated temperature sensors at surface weather stations reveal systematic measurement errors, due primarily to the uncompensated common environmental variables of solar irradiance, surface albedo reflectance, and insufficient wind speed [6,8,31,33,41,165,166,167]. The systematic measurement error due to uncompensated environmental variables is examined next.

#### 3.3.1. De Bilt (Netherlands)

Brandsma and van der Meulen reported extensive field-calibration experiments using platinum resistance thermometer (PRT) sensors mounted within nine naturally ventilated louvered sensor shields of varying configurations [168,169]. Air-temperature-measurement differences were calculated relative to a naturally ventilated KNMI multiplate reference. All the screens were equipped with PRTs of identical make. Thus, measurement errors are restricted to the impact of the shield. In general, the mean seasonal differences with respect to the KNMI reference was ≤0.1 °C. However, the question addressed here is measurement accuracy, rather than inter-screen means.

**Figure 5:**
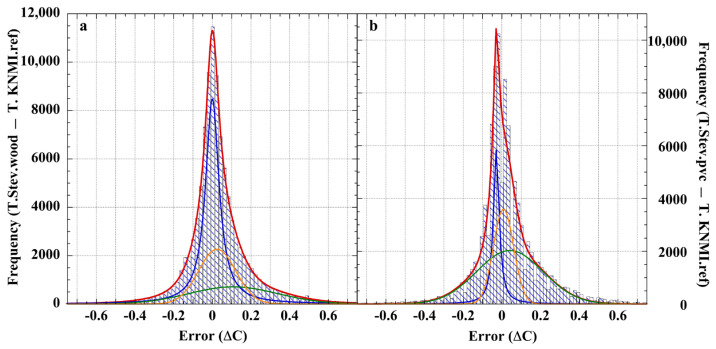
Histogram of calibration error magnitude versus frequency for a Pt500 PRT temperature sensor within a naturally ventilated: (**a**) wooden Stevenson screen (*N = 101,529*; RMS = ±0.20 °C)); or (**b**) a PVC Stevenson screen (*N = 99,973*; RMS = ±0.19 °C), each relative to the naturally ventilated KNMI reference screen [169]. Each fit (r^2^ = 0.999; 0.943, respectively) included a Lorentzian and two Gaussians: (red line), the fit; (blue line), the Lorentzian; (orange line), Gaussian one; and (green line), Gaussian two.

The test shields included two naturally ventilated Stevenson screens of KNMI design; one constructed of wood and the other of polyvinyl chloride (PVC). These are of particular interest because LiG thermometers housed in Stevenson screens have provided the great bulk of historical land-surface air temperatures [170]. Figure 5 shows the frequency distribution histograms of the (T_Stev_. minus T_KNMI_) temperature-measurement errors obtained within the wood or PVC Stevenson screens.

The error distributions are neither normal nor comparatively equivalent, and are biased warm. The overall error frequency maxima (*f_m_*) and RMS errors are similar (0.00 °C and 0.01 °C, resp.; RMS = ±0.2 °C). An adequate fit to each distribution required the combined intensities of a Lorentzian and two Gaussian lines (Table 3). These line shapes do not necessarily reflect physically real processes, but, rather, indicate the structural complexity of the measurement error. Although the fitted line shapes in each histogram are analogous, the intensities, FWHM, and offsets are disparate. Each screen was subjected to virtually identical external variables of wind speed and irradiance, but produced different internal thermal environments. The measurement errors clearly change with material state, as the screens were dimensionally equivalent.

**Figure 6:**
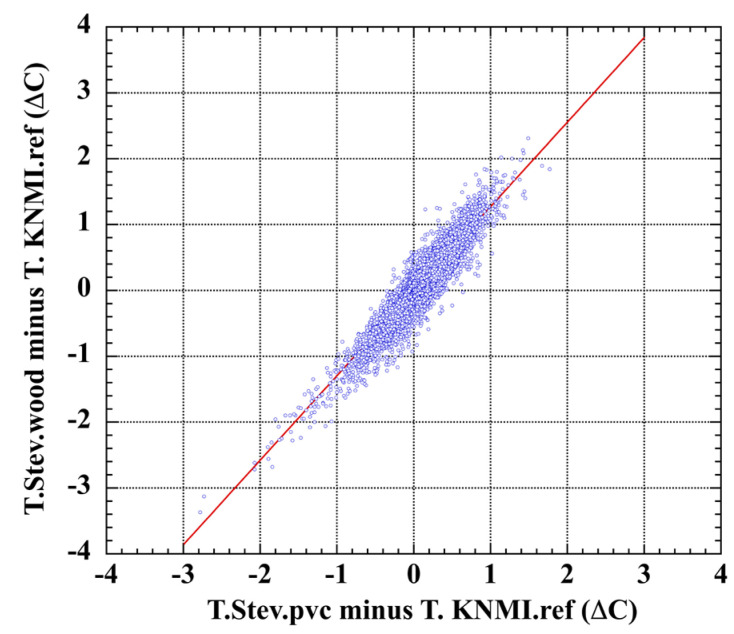
Correlation plot of systematic air-temperature-measurement error produced by PRT sensors in naturally ventilated wood or PVC Stevenson screens during January 1989–January 1990. Errors are relative to a PRT in the naturally ventilated KNMI reference screen, and derive from the thermal impact of irradiance or inadequate wind speed. The line is a least-squares fit: *y = (1.284 ± 0.002)x* − *(0.0126 ± 0.0005); correlation r = 0.92*.

A correlation plot of the calibration-error data sets for the wooden and the PVC Stevenson screens (Figure 6) shows correlation r = 0.92, which strongly disconfirms the universal assumption of random measurement error in air-temperature measurements. Analogous calibration error histograms and correlation plots for the De Bilt Socrima, R. M. Young, and Vaisala screens are shown in Figure S1 of the Supplementary Materials.

Mean pair-wise correlations of systematic error were assessed for all the experimental screens of the Brandsma–van der Muelen test series (Table 4). Five pairs show error correlation r ≥ 0.5 and another 12 show r ≥ 0.25.

Significant screen-pair correlations of systematic error are also evident year-by-year over the seven-year test period, shown in Tables S1–S7 in the Supplementary Materials. The six-year composite r = 0.88 of the Stevenson screen pair was the strongest correlation of measurement error. This is especially relevant because the historical land-surface air-temperature record is dominated by LiG thermometers housed in Stevenson screens.

#### 3.3.2. Plaine Morte Glacier (Swiss Alps)

Huwald and associates installed a meteorological station on the Plaine Morte Glacier in the Swiss Alps (2700 m mean altitude) [36].

Test temperature sensors included a PRT mounted within a naturally ventilated R.M. Young multiplate shield and a fine-wire thermocouple. These instruments were calibrated against a sonic anemometer air-temperature reference, which is insensitive to irradiance and wind-speed effects.

**Figure 7:**
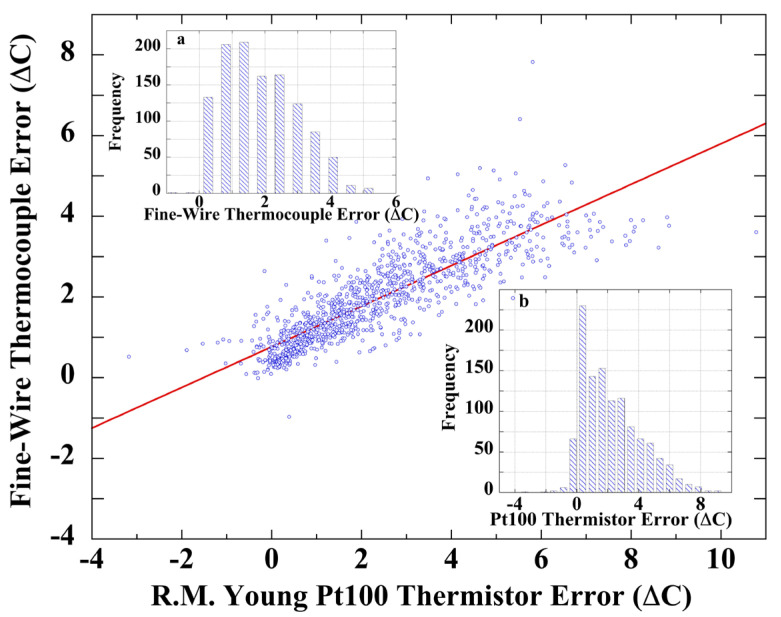
(points), Correlation plot of air-temperature-sensor calibration error (Alpine Plaine Morte Glacier, 2700 m, 8 February through 11 March 2008) of: a Pt100 thermistor in an R.M. Young multiplate shield versus the fine-wire thermocouple [36] relative to a sonic anemometer reference sensor (see text). (Line), linear least squares fit to the points: *y = (0.503 ± 0.009)x + (0.76 ± 0.03); correlation r = 0.86*. Insets: error-frequency histograms of: (**a**), the fine-wire thermocouple (1.9 ± 1.1 °C); and (**b**), the PT thermistor in the R.M. Young multiplate (2.2 ± 1.9 °C).

The naturally ventilated R.M. Young multiplate shield and the fine-wire thermocouple exhibited significant calibration errors; ε_calib_ = 2.2 ± 1.9 °C and ε_calib_ = 1.9 ± 1.1 °C, respectively. The correlation plot, Figure 7, yielded sensor-calibration measurement error correlation r = 0.86. The error-frequency histograms (Figure 7, insets) show that each error set strongly departs from a normal distribution. Shapiro–Wilk tests for normality yielded: fine-wire thermocouple error, W(1154) = 0.941, *p* < 0.001; and PT100 thermistor error, W(1155) = 0.959, *p* < 0.001, indicating non-normal distributions and confirming the visual appraisal.

#### 3.3.3. HOBO Thermistors, Ottawa

Mauder and associates reported a calibration of 25 air-temperature sensors, each consisting of a thermistor housed in a naturally ventilated multiplate HOBO shield [90]. The reference temperature-measurement standard was a high-accuracy thermistor housed within a fan-aspirated radiation shield.

**Figure 8:**
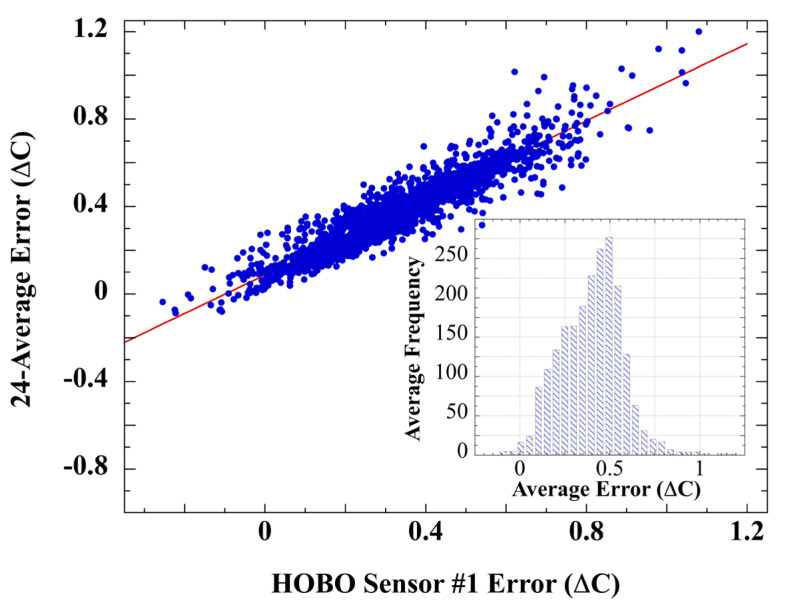
(HOBO minus reference) sensor systematic measurement error. (Points), 24-error average versus the error of HOBO sensor #1 (of 25). (Line), linear least-squares fit, *y = (0.881 ± 0.007)x + (0.087 ± 0.003); correlation r = 0.94*. Inset: Histogram of the complete 25-error average. RMS ε_avg_ = ±0.43 °C; mean offset = 0.40 °C. Shapiro–Wilk W(2160) = 0.989, *p* < 0.001, indicating non-normality.

The 25 HOBO sensors were arranged in a 5 × 5 grid situated in a 10 m × 10 m area. The reference thermistor was located 2 m away from one corner of the grid. Calibration air temperatures were measured during 2–3 May 2007. Calibration error for each HOBO sensor is the temperature-measurement difference with the aspirated reference sensor. Figure 8 displays the calibration error mean of HOBO #2 through HOBO #25 plotted against the error produced by the HOBO #1 thermistor.

The very high r = 0.94 correlation of HOBO #1 error with the 24-average is pair-wise repeated for the entire set of 25 HOBO sensors. The highly non-normal error distribution (Figure 8, inset) is likewise evident in each of the 25 HOBO error series (Figure S2 in the Supplementary Materials). Table S8 in the Supplementary Materials provides RMS calibration error for all 25 HOBO shields, the individual pair-wise inter-sensor error correlations, and the results of Shapiro–Wilk tests for normality. The HOBO error means and calibrations are highly correlated (r = 0.97, Figure S3 of the Supplementary Materials), indicating that the 25 HOBO shields produced very similar measurement errors in response to heating from irradiance and insufficient wind speed. The combined HOBO error set (*N = 54,000*) exhibited a non-normal distribution (Figure S4 in the Supplementary Materials), all of which again disconfirm the notion of random measurement error.

#### 3.3.4. Wire Thermocouples, SRNL

**Figure 9:**
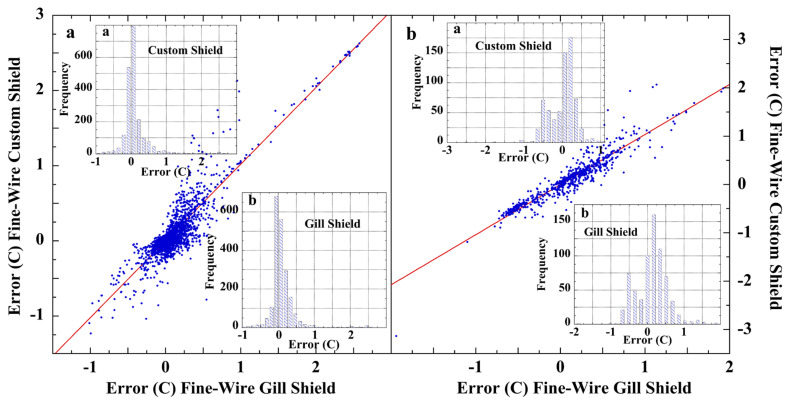
(Points), (test minus calibration) error-correlation plots of fine-wire thermocouple sensors in a naturally ventilated Gill shield or a naturally ventilated custom plate shield: (**a**), calibration reference was a fine-wire thermocouple in an aspirated Yankee 2010 shield; (**b**), calibration reference was a PRT in an aspirated MetOne 327-C instrument. (Red lines), linear least-square fits to the points: (**a**), *y = (1.02 ± 0.01)x − (0.003 ± 0.004) correlation r = 0.90*; (**b**), *y = (1.04 ± 0.01)x − (0.004 ± 0.007); correlation r = 0.94*. (**a**,**b**) insets: histograms of the sensor-calibration error.

An extensive field test of fine-wire thermocouple temperature sensors was carried out in 2008 at the Savannah River National Laboratory [171]. The purpose was to test the accuracy of air-temperature measurements using sensors housed in unaspirated shields. Two reference sensors included a fine-wire thermocouple and a platinum resistance thermometer (PRT) each housed in a Yankee MetOne-2010 aspirated shield and accurate to ±0.1 °C. The two test sensors were fine-wire thermocouples, with one in a naturally ventilated Gill shield and the other within a naturally ventilated custom multi-plate shield having the same spacings as the test Gill shield. Figure 9 shows that highly correlated measurement errors were produced by the two naturally ventilated fine-wire sensors, when differenced against either of the two aspirated reference sensors.

In Figure 9, the error distributions and the mean magnitudes varied with the aspirated calibration sensor (Table 5). This disparity was assigned to air-flow differences, with the aspirated thermocouple yielding the more accurate calibration [171]. Nevertheless, each calibration sensor revealed that the naturally ventilated screens produced correlated systematic measurement errors. The Shapiro–Wilk test (Table 5) indicated the measurement errors were non-normal. Similar correlated or non-random errors were found in other co-located air-temperature sensors, including those on a floating buoy, as shown in Figures S5–S8 and Table S9 in the Supplementary Materials [67].

All the examined calibration-experiment error sets revealed a warm bias and a non-normal distribution. Additional calibration experiments, not discussed here, have yielded similar air-temperature-measurement uncertainties that invariably arise with the use of naturally ventilated shields [34,35,44,45,68]. A high-quality Siemens thermistor housed in a naturally ventilated Stevenson screen produced a non-normally distributed measurement error (*N = 144*) (Figure S9 in the Supplementary Materials)) [35]. In the same experiment, copper–Constantan wire thermocouple sensors produced highly correlated air-temperature-measurement errors. Likewise, calibration of a PRT housed in a MetSpec double-louvered plastic Stevenson screen produced a non-normal distribution of systematic measurement error (*N = 81,504*) (Figure S10 of the Supplementary Materials) [45].

Table 6 lists the uncertainties due to systematic measurement error as produced by naturally ventilated shields during the several field-calibration experiments described above.

The full KNMI field-calibration experiments (Section 3.3.1) permit a test of the distribution produced by multi-sensor cumulated measurement errors in the land-surface global air-temperature record [167,168,169]. This field test consisted of five naturally ventilated PRT sensors, including two Stevenson screens (constructed from wood or PVC), and Visalia, Socrima, and T. Young multiplate shelters. Each screen was exposed to at least two years of varying weather, across the six years of the field calibration (January 1989–February 1995). The large sample size, varying weather, and multiple screen types in combination provided a valid test of the assumption of strictly random errors in land-surface temperature measurements. The assumption requires that a large varied error data set produces a normal distribution.

**Figure 10:**
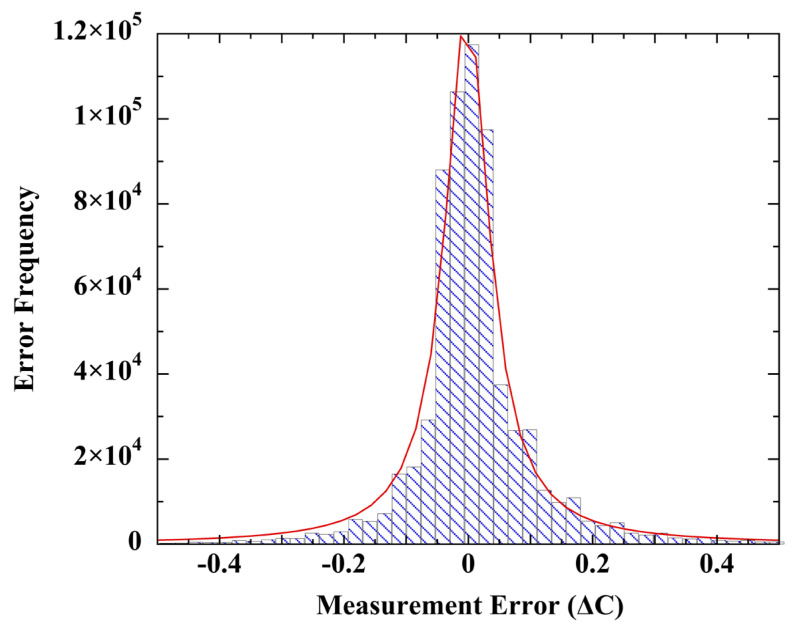
Histogram of cumulated measurement errors (*N = 667,403*) arising within five naturally ventilated screens during the De Bilt six-year field-calibration experiment. The red line is a Lorentzian fit (*r^2^ = 0.98, Γ = 0.086 ± 0.001; x_0_ = 0.0018 ± 0.0005*). The error range = −4.01 °C to 5.27 °C, with RMSE = ±0.12 °C.

For this test, the complete set of (sensor minus KNMI reference) calibration errors from all five sensors were appended into a single file (*N = 667,403*). Figure 10 shows a histogram of the combined error data points, which was well fitted with a Lorentzian line-shape. A Gaussian fit (Figure S11 in the Supplementary Materials) was notably deficient at the wings. The near-Lorentzian distribution and the evidence of correlated error strongly disconfirm the notion of random measurement error.

### 3.4. Sea-Surface Temperature

Categorical sea-surface temperature (SST) can be divided into *T_t_*, the physically correct (true) temperature of the in situ waters, *T_s_*, the temperature of an acquired water sample, and *T_m_*, the measured temperature. *T_t_* is generally unknown absent in situ measurement using a high-accuracy, high-precision temperature sensor. Under ideal circumstances, *T_s_* ≈ *T_t_* within sampling integrity, and *T_m_* ≈ *T_s_* within the accuracy limit of a standard sensor—historically, a LiG thermometer or, less often, a thermistor—and given care in measurement protocol. For example, a U.S. Naval study of the reliability of expendable bathythermograph (XBT) measurements, carried out by trained personnel reported LiG thermometer bucket SSTs to be within 0.01 ± 0.1 °C of the measurements of a highly accurate conductivity/depth/temperature (CDT) sensor [173].

#### 3.4.1. Context

Shipboard measurements prior to year 1990 contributed the great bulk of sea-surface temperatures (SSTs) entering the International Comprehensive Ocean–Atmosphere Data Set (ICOADS) [174,175,176,177,178]. The ICOADS compilation is used in the construction of the global air-temperature record [13,55,59,72,75,179]. The great bulk of shipboard SST measurements utilized LiG thermometers to measure the temperature of either engine-intake water or of a seawater sample drawn up onto deck using a specialized meteorological bucket [75,176,180]. The accuracy of the historical measurements is under examination here.

The sources and estimates of measurement errors attending bucket and engine-intake SSTs have been thoroughly discussed elsewhere and will not be reiterated here [74,75,79,80,82,84,181,182,183,184].

The limits of resolution and non-linearity specific to LiG thermometers, described in Section 3.1.1 and Section 3.1.2 above, apply equally to LiG thermometers used to measure sea-surface temperature (SST) from bucket samples and ship-engine-intake water. Likewise, the uncertainty due to Joule-drift will apply to those SSTs measured using LiG thermometers manufactured prior to 1890.

Compilations of the global SST record are presently conducted under the assumption that the measurement error on each ship (platform) takes a random distribution about a constant ship mean error, relative to the physically correct SST. The global set of ship error means is further assumed to be randomly distributed [71,73,185]. These assumptions persist despite published reports that SST measurement errors vary with the ship, with the cruise, and with the crew [79,80,81]. Nevertheless, the historical SST measurement errors are assumed to coalesce into a normal distribution about their global average mean error offset, which can be removed by differencing. This, in turn, is taken to justify reducing global mean SST measurement error, ±σ_m_, by 1/$\sqrt{N}$ in the global record, rendering SST measurement uncertainty insignificant.

#### 3.4.2. Does Semivariogram Analysis Yield the SST Measurement Error Mean?

The semivariogram regression has been used to extract the mean of measurement error from the historical SST record [71,185,186,187]. With subtraction of the derived global mean offset, the residuum of error is assumed to be a normal distribution with a mean of zero and a final negligible uncertainty determined as ±σ/$\sqrt{N}$.

The variogram method is derived from Geostatistics and is used to examine the behavior of paired observables in a spatial field [188,189]. Differences in the magnitude of some set of field observables ‘*x*’ (e.g., soil pH) are assumed to depend only on the separation distance, typically ‘*h*’. The variogram tracks the square of the differences:

(1) $$2\gamma \left(h\right)={\left[\left(x+h\right)-\left(x\right)\right]}^{2},$$

where *x + h* is the measurement magnitude of observable *x* at a distance *h* from any reference measurement of *x*, and [(*x + h*) − (*x*)]^2^ → 0 *as h* → *0*. Squaring ensures that 2*γ*(*h*) is always positive as *h* → 0. If, on the other hand, 2γ(h) ≠ 0 at *h* = *0,* then the ordinate offset, termed the *nugget*, derives from measurement error, *ε_m_*, plus microscale variability, c_MS_ [188]. Kent and colleagues (cited above) used semivariogram analysis, ½[2*γ*(*h*)], to appraise pairs of historical SSTs that had been simultaneously measured on spatially separated ships, with the inter-ship distance ranging from proximate to 300 km [71,186]. Microscale SST variability, c_MS_, was set to zero at closest proximity. Thus, [(*SST*_1_) − (*SST*_2_)]*^2^* regressed against ship-separation distance (*h*) yielded the *nugget* at *h* = 0 as a positive offset. The *nugget* was taken to be twice the variance of ship-measurement error global mean, and $\frac{1}{2}\sqrt{nugget}=\pm \varepsilon_{m}$. Semivariogram analysis was, likewise, used to estimate the error mean in historical marine wind-speed measurements [190].

Examining this usage, any SST measurement *T_m_* = *T_t_ + ε_m_*, where *T_t_* is the physically correct (true) temperature of the in situ ocean water and *ε_m_* is the combined systematic and random measurement errors. In any such measurement, *T_t_* and *ε_m_* are completely convolved. The correct magnitude of either quantity is not known. When two ships are spatially separated, *SST_t_*_1_ *≠ SST_t_*_2_ and *SST_m_*_1_ − *SST_m_*_2_ = (*T_t_*_1_ *+ ε_m_*_1_) − (*T_t_*_2_ *+ ε_m_*_2_) = Δ*T_t_*_1,2_ + Δ*ε_m_*_1,2_. When *h* = 0, *T_t_*_1_ = *T_t_*_2_, Δ*T_t_*_1,2_ = 0, and *SST_m_*_1_ − *SST_m_*_2_ = Δ*ε_m_*_1,2_. Thus, variogram SST analysis regresses the convolved (Δ*T_t_*_1,2_ + Δ*ε_m_*_1,2_)^2^ against *h*, and the *nugget* offset at *h* = 0 is (Δ*ε_m_*_1,2_)^2^ because Δ*T_t_*_1,2_ = 0. That is, the variogram *nugget* is the square of the estimated mean *difference* in error, not the square of the estimated mean error.

In constructing the *semi*variogram from the variogram, this *nugget* has been divided by two on the grounds that it represents the mean error of two ships [186,190]. However, as the *nugget* is, in fact, a difference of errors, dividing by two is incorrect; a difference of one ship has no discrete meaning. Thus, the $\sqrt{{\left(nugget\right)}^{2}}=\sqrt{{\left(\Delta \varepsilon_{m1,2}\right)}^{2}}=\pm \Delta \varepsilon_{m1,2}$ is the estimated root–mean–square (RMS) of the SST measurement error *difference* mean. It is not the estimated RMS of the SST measurement error mean itself. The magnitude of the SST error mean itself remains unknown. Only the mean difference is revealed. Thus, the mean error of historical ship SST measurements itself remains unknown (and likely unknowable). Homologously, variogram analysis of the marine-wind-measurement error yielded the mean error difference, not twice the error mean [190]. The argument, thus, requires revision.

#### 3.4.3. Are SST Measurement Errors Random?

##### Instrumental Calibration

The assumption of random error covers the methodologically independent sets of LiG SST measurements arising from buckets or engine-intakes. Field calibrations of bucket and engine-intake SSTs were carried out by Charles Brooks aboard the *R. M. S. Empress of Britain* cruise ship and aboard the oceanographic research ship *C.F.A.V. Endeavor* [79,81,191].

**Figure 11:**
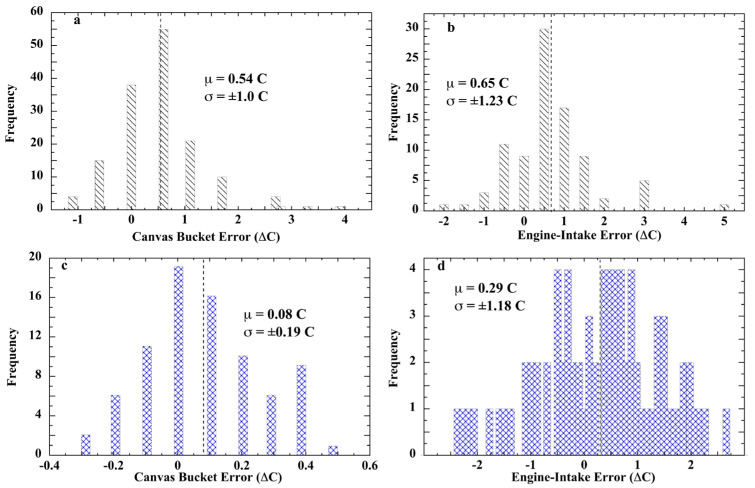
Frequency histogram of SST measurement error revealed by field-calibration experiments of Brooks: (**a**) bucket (*N = 150*); (**b**) engine-intake (*N = 80*); tin bucket reference sample [193]. The experiments reported by Tabata, (**c**) bucket (*N = 80*) or (**d**) engine-intake (*N = 54*). Salinity–temperature–depth recorder (S.T.D.) reference [81]. Dashed vertical lines mark the arithmetic mean value.

The tin-bucket reference thermometer used by Brooks was graduated in 0.5 °C divisions and calibrated as accurate to ±0.2 °C [192]. The *R.M.S. Empress of Britain* engine-intake thermometer was also scored to 0.5 °C and estimated to be of ±0.3 °C precision. In the later experiments aboard the *C.F.A.V. Endeavor* as reported by Tabata, a salinity–temperature–depth (STD) recorder, accurate to ±0.02 °C, provided the calibration-reference temperatures. The results of the Brooks and the Tabata calibration experiments are displayed in Figure 11.

The error distribution of measurements acquired aboard the *C.F.A.V. Endeavor* research vessel (Figure 11c) shows that bucket SSTs can be accurate to ±0.2 °C in the hands of trained personnel [81,191]. On the *R.M.S. Empress of Britain*, however, bucket error was much larger and varied with the watch; an outcome also noted by Saur (*cf. 4-Saur* below). Visual inspection indicates their divergence from Gaussian distributions.

Each of the engine-intake calibrations yielded a bias and uncertainty of 0.3 ± 1.2 °C (1σ), which obviates accuracy. Tabata rejected engine-room heat as the source of positive bias because a large fraction of readings was lower than the STD reference temperature. Instead, he assigned the ±1.2 °C standard deviation to reading error. However, carelessness in thermometer reading by professionals and crew aboard a meteorological-research vessel does not seem likely.

Nevertheless, the ship bucket and engine-intake measurement errors displayed non-normal distributions, inconsistent with random error. These calibration outcomes are tested more widely below.

##### The Difference of Normal Distributions

The assumption of random measurement error is subject to a general test. Any normally distributed data set X = [x_1_, x_2_, … x_n_] can be expressed in its standardized form, Z = (X − µ)/σ, where Z = [z_1_, z_2_, … z_n_] are real numbers and each x_i_ is z_i_ standard deviations away from µ [194]. Rearranging, X = Zσ + µ and the difference between two normally distributed data sets is X_2_ − X_1_ = ΔX_2,1_ = (Zσ_2_ + µ_2_) − (Zσ_1_ + µ_1_) = Z($\sqrt{\left(\sigma_{2}^{2}-\sigma_{1}^{2}\right)}$ + (µ_2_ − µ_1_). Then ΔX_2,1_ = ZΔσ_2,1_ + Δµ_2,1_ and the distribution of the differences is Z = (ΔX_2,1_ − Δµ_2,1_)/Δσ_2,1_. Thus, the difference of two normal distributions is another normal distribution. If the respective canvas bucket and engine-intake SST measurement errors are normally distributed, therefore, their difference set should be normally distributed. This approach has the advantage that error distributions can be illuminated through error difference sets, without needing to know the errors themselves. This test follows.

##### Bucket SSTs

**Figure 12:**
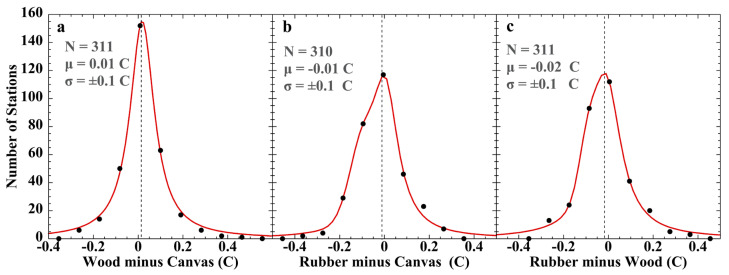
(Points) Histogram of the frequency of inter-bucket differences of SST measurement error, $\Delta \varepsilon_{mb_{1,2}}$, when using a wood, canvas, or rubber meteorological bucket [195]. (Red lines): (**a**) Lorentzian fit; (**b**,**c**) combined Lorentzian plus Gaussian fits (Fit r^2^: (**a**) 0.996; (**b**) 0.994; (**c**) 0.994). The empirical mean (µ, vertical dashed line) and standard deviation (σ) are on the face of each panel. The means are off-maximum because the distributions are skewed. A single difference point at 0.7 °C was excluded from panel **b**, leaving 310 points.

In a unique experiment, researchers aboard the Sea Education Association (SEA) research vessel *SSV Robert C. Seamans* measured three sets of near-simultaneous SSTs during the S-217 transect across tropical waters, using a traditional wooden bucket (~8 L), a general-purpose ship’s canvas bucket (~11.5 L), or a meteorological rubber bucket (~0.7 L) [195]. Hourly casts using each bucket were conducted consecutively over a period of about 5 min. SSTs were measured using a traceable thermistor sensor accurate to ±0.1 °C. The three buckets yielded statistically indistinguishable SSTs, with a mean difference of 0.0 ± 0.1 °C. Given simultaneous measurements of the same waters, *T_sw_* = *T_sc_* = *T_sr_* and *T_mb_* = *T_s_ + ε_mb_*, where subscript ‘*s*’ is sample, ‘*w*’, ‘*c*’, or ‘*r*’ subscripts designate wood, canvas, or rubber, respectively, ‘*b*’ indicates bucket, and *ε_m_* is total measurement error. Then differencing, e.g., the wood and canvas bucket measured SSTs, (*T_mw_* − *T_mc_*) = (*T_s_ + ε_mw_*) − (*T_s_ + ε_mc_*) = (*T_s_* − *T_s_*) *+* (*ε_mw_* − *ε_mc_*) = Δ*ε_mw,c_*. That is, differencing any two of the bucket SST measurements yields the difference of the measurement errors. If each bucket-measurement error is random and each *ε_m_* is normally distributed, then each of the three possible Δ*ε_m_* difference sets should also be normally distributed. Figure 12 shows the results of this test.

Shapiro–Wilk normality tests of the three inter-bucket error-difference data sets yielded: wood minus canvas, W(311) = 0.891, *p* < 0.001; rubber minus canvas, W(311) = 0.916, *p* < 0.001; and rubber minus wood, W(311) = 0.926, *p* < 0.001. Each result is consistent with non-normal inter-bucket difference errors.

The best fit to the histogram points was obtained using a Lorentzian line (Figure 12a), or a Lorentzian and Gaussian in combination (Figure 12b,c). The latter results do not necessarily indicate biphasic errors. Test fits of each $\Delta \varepsilon_{mb_{1,2}}$ histogram with a single Lorentzian or single Gaussian (Figure S11 of the Supplementary Materials) indicated none of them are consistent with a normal distribution. Maximally, no more than one of the three originating *ε_mb_* sets can be random error.

##### Engine-Intake SSTs

The test for random error can be extended to differences between ship engine-intake and bucket SSTs. The physically correct temperature, *SST_t_*, can vary with the sample acquisition depth [173,195,196,197]. However, surface mixing from wind and/or wave action can homogenize the thermocline [79,87,173,196,198,199]. Thus, the temperature of the engine-intake sample, *T_si_*, and of the bucket sample, *T_sb_*, may be similar or may differ, depending upon environmental variables. Despite the negative thermocline gradient, however, engine-intake SSTs average about 0.3 °C warmer than bucket SSTs [183,186,200].

If the thermocline is mixed into homogeneity, then *T_si_* = *T_sb_*, and $\Delta T_{s_{i,b}}=\Delta \varepsilon_{m_{i,b}}$ so that the analysis under The Difference of Normal Distributions applies. To appraise the most general case, thermal non-equivalence of bucket and engine-intake seawater samples is assumed. Then *T_si_* ≠ *T_sb_*, *T_mi_* = (*T_si_ + ε_i_*) and *T_mb_* = (*T_sb_ + ε_b_*). Finally, *T_mi_* − *T_mb_* = (*T_si_ + ε_mi_*) − (*T_mb_ + ε_mb_*) = (*T_mi_* − *T_mb_*) *+* (*ε_mi_* − *ε_mb_*) = Δ*T_b,I_ +* Δ*ε_m_*_(*b,i*)_, where subscripts *b* and *i* refer to bucket and engine-intake samples, respectively. The differences then yield the distribution of Δ*ε_m_*_(*b,i*)_*,* but having a mean offset due to the contribution of Δ*T_b,i_*. This condition is examined next.

1. Brooks

**Figure 13:**
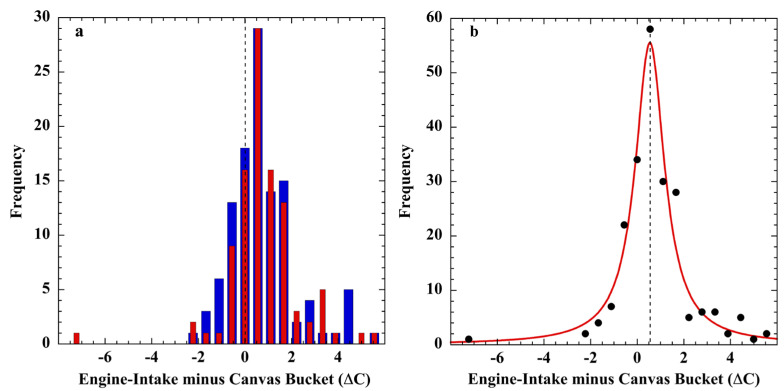
(**a**) Histograms of engine-intake minus canvas bucket SST measurement differences for each of two 1924 West Indies cruises of the *R.M.S. Empress of Britain*: (blue bars), 18 January–20 February 1924, *N = 113, µ= 0.76 °C, σ = ±1.6 °C*; and (red bars), 23 February–23 March 1924, *N = 101, µ = 0.79 °C, σ = ±1.7 °C*. The red bars have been narrowed to improve visualization. (**b**) (points), combined bucket minus intake differences from the same two West Indies cruises; (red line), Lorentzian fit (*Γ = 1.53, x_0_ = 0.55, r^2^ = 0.95*).

Figure 13 shows the SST (*T_i_* − *T_b_*) = Δ*T_b,I_ +* Δ*ε_m_*_(*b,i*)_ distribution (*N = 214*) Charles Brooks obtained aboard the *R.M.S. Empress of Britain* during January–March 1924 during two West Indies cruises (9°–35° N latitude), each along nearly the same track [79].

Neither set of measurement differences nor the combined difference data set are normally distributed (Figure 13a,b, *N = 214*). The combined errors (Figure 13b) appear to have coalesced into a Lorentzian-like distribution similar to the land-surface temperature-measurement errors described above.

2. WMO

During 1968–1970, the World Meteorological Organization (WMO) Working Group on Technical Problems of the Commission for Marine Meteorology carried out a program to establish the structure of (*T_i_* − *T_b_*) SST differences [183].

**Figure 14:**
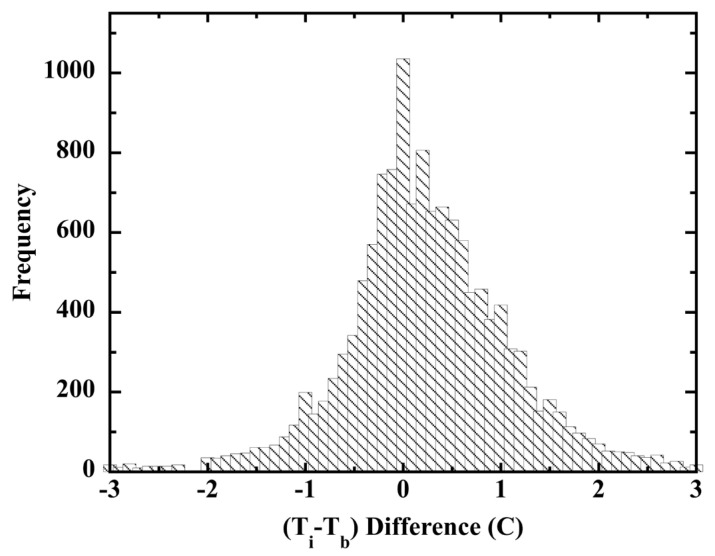
Histogram of the frequency distribution of engine-intake minus bucket SST measurement differences (*N = 13,511*, arithmetic *μ = 0.24* °C, *σ = ±0.77* °C) from Table III of ref. [183].

The majority of the SSTs were measured in the major ocean basins between latitudes 50° N and 50° S, but with 11% of the measurements at latitudes >50°. Routine shipboard measurement methods were employed and instruments (primarily mercury LiG thermometers) were calibrated before each voyage. Ship crew-members took measurements at standard times, using the calibrated instruments. Of a total 16,132 observer logs, 13,876 included simultaneous bucket and engine-intake SST measurements. These were acquired on at least five classes of ship under all manner of weather, wind, and cloud cover. Figure 14 is a histogram of the 13,511 *T_i_* − *T_b_* differences occurring within the ±3 °C range ([183]; Table 3).

The ship-board officers recruited into the WMO project likely carried out their protocols and observations with more care than common in the voluntary observing ships (VOS) program. In this event, the WMO (*T_i_* − *T_b_*) data set provides a reasonable estimate of a lower limit of (engine-intake)−(bucket) measurement error differences in the global record.

The (*T_i_* − *T_b_*) histogram in Figure 14 does not present the normal distribution expected for the differences of random measurement errors. The complex distribution was best fit with the sum of three Lorentzians (Figure S12 in the Supplementary Materials). A fit with three Gaussians was slightly poorer, but the fit serially improved with each substituted Lorentzian.

3. Walden

Walden reported 13,847 (*T_b_* − *T_E_*) differences of almost simultaneous bucket (*T_b_*) and engine-intake (*T_E_*) SST measurements, principally carried out on German merchant ships during the early 1960s [198]. Measurement differences were categorized by latitudinal bands (0° to >55° N, S) and wind speed.

**Figure 15:**
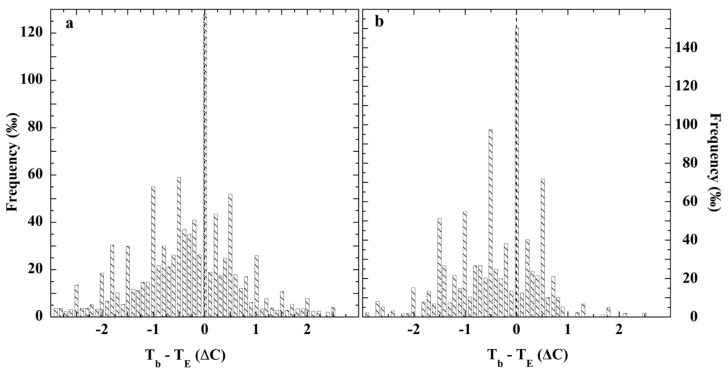
Frequency of bucket (T_b_) minus engine-intake (T_E_) SST differences at different ranges of wind speed for N, S latitude 25° to 49.9°, over the range ±3 °C. Outliers >|3| °C were excluded. (**a**): Wind speed 5–7 Bft; ΔT = −0.3 ± 1.5 °C. (**b**): Wind speed ≥8 Bft; ΔT = −0.5 ± 1.5 °C. (1 Beaufort = 0.836 m/s).

Figure 15 displays the (*T_b_* − *T_E_*) differences as reported by Walden for German ship SSTs measured over 25–49.9° North and South latitudes under wind speeds of 5–7 Bft and ≥8 Bft (1 Beaufort = 0.836 m/s) [198]. Global mean wind speed over the oceans is about 7.4 m/s (8.8 Bft), ranging about 1 m/s less than average in the tropics and about 1 m/s more in the 25°–75° N, S latitudes [201]. Thus, Figure 15 provides a good global estimate of typical bucket-intake SST measurement differences. Neither difference data set exhibits the normal distribution expected from random errors. The 0–1 Bft and 2–4 Bft (*T_b_* − *T_E_*) difference distributions reported by Walden are also non-normal.

The remaining *T_b −_ T_E_* determinations were: 0–1 Bft, Δ*T = −0.3 ± 1.2 °C* and for 2–4 Bft, Δ*T = −0.4 ± 1.3 °C*. In every case, bucket temperatures were cooler than those recorded from engine-intake thermometers, despite the marine thermocline. Recombination of the published latitudinal (*T_b_* − *T_E_*) differences into a single-difference data set representing 25–49.9° N, S and all wind speeds yielded a non-normal distribution of global coverage (Figure S13 of the Supplementary Materials). Thus, regionally and globally, the *T_b_* − *T_E_* difference frequency histograms exhibited non-normal distributions, disconfirming the notion of random SST measurement error.

4. Saur

**Figure 16:**
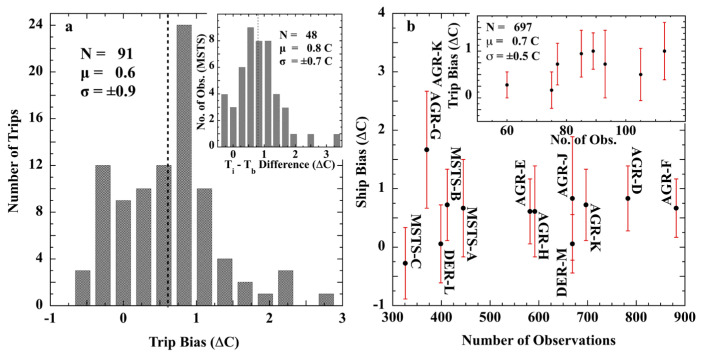
(**a**), Histogram of the combined (*T_i_* − *T_b_*) mean biases of all 12 military ships after 91 trips extending over about a year. Inset: Histogram of (*T_i_* − *T_b_*) for a single trip of an MSTS vessel. The dashed lines locate the mean. (**b**), The aggregate means (points) and standard deviations (whiskers) of (*T_i_* − *T_b_*) for each of the 12 military ships. Inset: the bias means (points) and their standard deviations (whiskers) for each of the eight trips of radar picket ship AGR-K, over August 1960–October 1961. MSTS is Military Ship Transport Service, AGR designates a radar picket ship, and DER indicates destroyer escort.

Saur reported 6826 engine-intake (*T_i_*) minus bucket (*T_b_*) SST differences obtained during experiments conducted on 12 U.S. military ships [80]. Three were military transport ships (MSTS) sailing trans-Pacific routes between May 1959 through May 1960. Nine were radar picket ships (AGR) stationed 300 mi (186 km) off the west coast of the U.S. during September 1960–January 1962. Specialty thermometers for bucket measurements were supplied to each ship, graduated to 0.2 °F (0.1 °C), readable to 0.1 °F (0.06 °C), and were accurate to at least ±0.15 °F (0.08 °C) following calibration. The buckets were of Scripps Institute of Oceanography design, and trained personnel carried out the bucket SST measurements. The engine-intake measurements were recorded by the ship crew in routine fashion, yielding SSTs representative of typical accuracy. The intake thermometers were to ship standard, noted to have 2 °F (1.1 °C) or, occasionally, 5 °F (2.8 °C) scoring. Ship bias (μ) and standard deviation (σ) of (*T_i_ − T_b_*) were calculated for each trip and each ship. Following the analysis under Difference of Normal Distributions, the assumption of random errors requires that the (*T_i_ − T_b_*) differences display a normal distribution. Figure 16 shows the results of this experiment.

Figure 16a is a histogram of combined mean biases for all 6826 paired (*T_i_* − *T_b_*) differences acquired during 91 trips of the 12 military ships. Coalescence into a normal distribution is not in evidence. Such a coalescence is required by the assumptions of a constant distribution of random error per ship and random error means across ships. Its absence disconfirms the assumption of random measurement error. The (*T_i_* − *T_b_*) distribution of the single MSTS ship, (Figure 16, inset), is visually inconsistent with normality. Saur described the trip statistics as, “*a typical distribution of the differences,* Δ*, from one trip of an MSTS ship*”, indicating (*T_i_* − *T_b_*) differences did not produce a normal distribution for any trip of any ship.

Figure 16b displays the scatter of (*T_i_* − *T_b_*) means among the ships while the whiskers indicate the scatter of the trip means of each ship. Figure 16b inset shows the scatter of the (*T_i_* − *T_b_*) means for eight trips of radar picket ship AGR-K. The (*T_i_* − *T_b_*) means varied from trip to trip for a single ship. Saur’s experiment indicates that none of the 91 single trip distributions of (*T_i_* − *T_b_*) were normal, nor was their aggregate normally distributed.

In recognition of this state, Saur concluded that, “*without improved quality control, the sea-surface temperature data reported currently and in the past are for the most part adequate only for general climatological studies …. If ship biases can be determined and corrections applied to existing sea water temperature records, it is estimated that the standard deviation of differences would be reduced to 1.3 °F (0.72 °C)*”. Saur’s judgment corroborates the findings of the WMO, of Walden, and of Brooks, and is applicable to the entire SST record prior to 1963. Even were it possible to remove a mean bias (presuming the value may be determined), the 1σ = ±1.3 F (±0.72 °C) uncertainty would remain in SSTs because the non-normal (T_i_ − T_b_) distributions indicate the random error assumption is violated and the statistical $1/\sqrt{N}$ rule is not applicable. SST errors do not average away.

Saur also discussed the accuracy experiment that Margaret K. Robinson reported and carried out aboard five U.S. Coast Guard weather ships [202]. Saur’s description of the Robinson experiment is directly relevant to the notion of SST measurement accuracy: “*[T]he injection thermometers were demounted and checked at several temperatures against an accurate standard thermometer. Temperature errors from the five thermometers ranged from −2.0 °F to 1.9 °F. Results of other observations indicated that the difference between injection temperature corrected for thermometer error and surface temperature “varied erratically both among ships and on individual ships at different speeds”*.”

Both Saur’s results and Robinson’s confute the assumption of random measurement error, and disconfirm the notions of constant error distributions per platform and of random error means between platforms. SST measurement error, thus, cannot be taken to reduce as $1/\sqrt{N}$.

#### 3.4.4. T_sample_ and T_true_

The relationship between *T_s_*—temperature of the water sample—and *T_t_*—the physically correct (“true”) in situ water temperature—was examined by Stevenson in an extended experiment carried out aboard the *Velero IV* research vessel, operated by the University of Southern California until 1985 [203]. SSTs were simultaneously measured aboard the ship and from a launch that systematically sampled nearby waters about the *Velero IV*. Duplicate sets of calibrated thermometers (for SST) and psychrometers (for air temperature) were used. SSTs were measured while the *Velero IV* was stationary or cruising, and either broadside or into the wind. The original experiment was extended to include bow-mounted thermistor probes to measure SST and air temperature while cruising. Bathythermograph (BT) casts were also carried out to evaluate bucket SSTs.

In the event, the ship was found to disturb surrounding waters under all conditions, occasionally out to 150 ft (46 m) depending upon wind conditions. Bucket SSTs measured from the *Velero IV* averaged about 0.5 °F (0.3 °C) cooler than the SSTs simultaneously measured from the launch. BT casts indicated bucket SSTs were consistent with the cooler water of the thermocline at 15–20 foot (4.6–6.1 m) depths. The bow-mounted probe gave reliable SSTs, but only when the *Velero IV* headed into the wind, or when cruising downwind at greater than wind velocity. Stevenson concluded that, “*The differences in water temperatures resulting from the presence of a ship will depend, to a considerable extent, on the temperature distribution in the upper layers. The occurrence of a thoroughly mixed layer extending well below the keel depth would preclude any significant changes in temperature caused by the ship. However, should there be a shallow thermocline disturbed by the vessel’s progress, modifications of water temperatures could be extreme, or even spectacular. One may then question the value of temperatures taken aboard a ship, or from any large structure at sea. Because the measurements vary with the wind velocity and the orientation of the ship with respect to the wind direction no factor can be applied to correct the data. It is likely that the temperatures are, therefore, useless for any but gross analyses of climatic factors, excepting, perhaps, those taken with a carefully-oriented probe*”.

The results indicated that a shipboard bucket seawater sample will not accurately convey the physically true sea-surface temperature unless three conditions are simultaneously true: (1) the thermocline is absent; (2) the vessel is heading into the wind; and (3) the mixing layer extends well below the depth of the keel. Thus, under nearly all conditions of the historical measurement record, *T_s_* ≠ *T_t_*, even when all due care was taken to protect an on-deck bucket sample from coincidental environmental impositions (primarily, wind) known to cause a temperature artefact.

That is, even when carefully executed by trained personnel, shipboard bucket SST measurements are likely to be several tenths of a Celsius offset from the physically correct SST. Although critically and centrally important, Stevenson’s experiment has received scant notice, and has not since been extended or used to qualify the global SST record. However, his conclusion confirms Saur, namely, that SSTs can contribute only to general climatological studies.

## 4. Discussion

This work has examined instrumental detection limits and systematic measurement errors hidden within land-surface-air and sea-surface temperatures. The impact of these measurement errors on the global air-temperature anomaly series is next discussed.

### 4.1. Land-Surface Air Temperatures

Air-temperature measurements contaminated with systematic error are indistinguishable from valid data. Systematically erroneous air-temperature trends will pass every statistical test used to validate a station record [7]. Harrison has noted that, “*Screen–air temperature differences represent systematic errors in air temperature measurements, which, unlike random errors, are not reduced by averaging*” and that “*… the overall prevalence of natural ventilation effects on screen temperature measurements seems unlikely to remain constant with time* [165]”.

Systematic measurement error is highly correlated among co-located naturally ventilated air-temperature sensors. This is not surprising in hindsight because the success of real-time filtering experiments has demonstrated the consistent impacts over time of radiant heating and wind speed [31,204]. Generalizing, naturally ventilated air-temperature sensors subject to similar solar heating and wind regimes will necessarily produce equivalently erroneous measurements.

Land-surface air-temperature measurements themselves are correlated across hundreds of km [19,65]. At 1200 km separation, mean correlation of anomalies is r ≈ 0.5 at latitudes > 23° and r ≈ 0.33 at latitudes < 23°. These teleconnections reflect coherently organized physical solar, wind, and precipitation phenomena within spatially extensive weather regimes [205,206,207,208,209]

Correlated weather in regional regimes ensures that widely distributed naturally ventilated land-surface air-temperature sensors will have correlated exposure to solar heating and wind effects, and, possibly, to homologous changes in local albedo. That is, multiple widely separated naturally ventilated sensors simultaneously exposed to a single overlying weather regime are effectively co-located. Though pairwise distant, they are exposed to equivalent environmental variables and, thus, to environmental impacts. On these grounds, it is proposed that systematic air-temperature-measurement error will be causally correlated across hundreds of kilometers in a manner strictly analogous to the correlation of air-temperature anomalies. Correlated systematic errors will be convolved within the correlated anomalies themselves. Cross-correlated measurement errors will be invisible within the cross-correlated anomalies.

Widely correlated systematic measurement error will not average away in a mean. Similarly, large data sets of the systematic air-temperature-measurement errors revealed in calibration experiments do not coalesce into normal distributions. There is no statistical demonstration that non-normal systematic measurement errors average away [30,210], particularly as the dimensions of error in the historical temperature record are entirely unknown.

Thus, the combination of experimental and observational grounds powerfully negates the assumption that air-temperature sensor-measurement error is exclusively random and uncorrelated. Correlated and non-normal systematic errors violate the assumptions of the central limit theorem, and disallow the statistical reduction of systematic measurement error as 1/$\sqrt{N}$. Only empirical confidence intervals from instrumental field calibrations are then available to condition measurements [211].

The proposed correlation of land-surface air-temperature sensor-measurement error across significant distances can be explicitly tested by installing calibration-competent aspirated and improved USCRN sensors adjacent to naturally ventilated USHCN sensors; most especially, those latter utilizing a LiG thermometer within a Stevenson screen [212]. The correlation of measurement errors produced by unaspirated USHCN Stevenson screens and/or MMTS shelters across arbitrary distances and topologies established by experiment can then be monitored. This experiment is recommended. Spatially correlated measurement error is expected on the above grounds.

Under these circumstances, sensor field-calibration experiments are strictly necessary to obtain the uncertainty bounds applicable to subsequent field air-temperature measurements. As noted here, few such experiments have been carried out. These, nevertheless, allow an estimate of mean systematic error in the global air-temperature record deriving from LiG thermometers in CRS and Stevenson screens and from MMTS temperature sensors.

### 4.2. Resolution Limits

The lower limit of resolution of LiG thermometers has been neglected during construction of the global air-temperature record. High-quality LiG thermometers scored to 1 °C or 1 °F per division have an instrumental lower limit of resolution 2σ = ±0.11 °C/°F. Qualifying this limit, Harrison noted that, “*Accuracy of LiG thermometers is typically ±0.2 °C, even though their resolution may be better, for example, with 0.1 °C divisions*” [93]. Fractional LiG temperatures smaller than the resolution limit have no physical meaning because they are not within the reliable detection capacity of the instrument. From Section 3.1.1, NIST calibrations of LiG thermometers and estimates of visual repeatability provide that, under ideal laboratory conditions, 2σ = ±0.33 °C/°F is the lower limit of uncertainty in any visually acquired temperature reading from a standard 1-degree/division LiG thermometer. Thus, the resolution-limited minimum uncertainty conditioning the LiG-derived air-temperature (not anomaly) record across the 20th century is, likewise, 2σ = ±0.33 °C/°F.

The non-linear thermal expansion of both mercury and ethanol, although a smaller component of measurement uncertainty, is presently uncorrected in the record. However, to the extent that structural knowledge of the LiG thermometers in use at 19th century surface stations and those used to compose earlier records is recoverable, correction for non-linearity may be possible.

Although the monthly average temperature error due to LiG nonlinearity of liquid expansion (Figure 1) will vary with the surface station and the season, its contribution to the uncertainty in global average temperature can be estimated. For this estimate, it is assumed that mercury and spirit thermometers are the sole sources of daily station maximum or minimum air temperatures, respectively, between 1900 and 1980. It is further assumed that uncertainty is distributed equally across the 0 °C ice-point calibration in a global average (Figure 1). The 1σ of uncertainty is taken to be 1/3 of the range of thermometer non-linearity. The total range shown in Figure 1 is considered to be 3σ about 0 °C. From Figure 1, the global average uncertainty due to non-linearity in a mercury-filled LiG thermometer is ±0.017 °C/°F (range ± 0.05°). For a spirit (ethanol-filled) LiG minimum thermometer the average uncertainty is ±0.33 °C/°F (range ± 1). This lower-limit estimate further assumes that 50% of all station temperatures were measured using a Fahrenheit thermometer, and that Celsius and Fahrenheit thermometers contributed equally to the global air-temperature record. The resulting estimate of global uncertainty from LiG non-linearity is shown in Table 7.

The complete minimal uncertainty in a global averaged land-surface air temperature through 1980 can now be derived. For the sake of the following estimate, all *T_max_* are assumed to be from mercury LiG thermometers, while all *T_min_* are assumed to derive from spirit LiG thermometers. From Table 7, the combined uncertainty in any daily mean land-surface air temperature due to non-linearity alone in LiG Celsius and Fahrenheit thermometers $2\sigma =1.96\times \sqrt{\left(0.00019+0.0713\right)/2}=\pm 0.371\;{}^{\circ}C$, for all *T_mean_* = (*T_min_* + *T_max_*)/2.

Spirit LiG thermometers provide about half the accuracy of the mercury LiG counterpart, yielding a per-measurement 1σ = ±0.309 C/°F (*cf.* Section 3.1.1, Table 1). The lower limit of uncertainty in any *T_min_* and *T_max_* land-surface air temperature prior to 1981 consists of the detection limit and the visual repeatability (*cf.* Table 1) combined in quadrature with the uncertainty due to non-linearity (Table 7). For spirit LiG *T_min_*, the merged Celsius and Fahrenheit uncertainty is,

(2) $$2\sigma \left(T_{min}^{spirit}\right)=1.96\times \sqrt{\left(0.5\times 0.309^{2}\right)+0.5\times {\left(0.556\times 0.309\right)}^{2}+\left(0.267^{2}\right)}=\pm 0.717\;{}^{\circ}C.$$

and for mercury LiG *T_max_*,

(3) $$2\sigma \left(T_{max}^{Hg}\right)=1.96\times \sqrt{0.5\times 0.166^{2}+0.5\times {\left(0.556\times 0.166\right)}^{2}+0.0138^{2}}=\pm 0.265$$

For *T_mean_* = (*T_max_* + *T_min_*)/2, the total uncertainty in the mean is given the *T_max_* and *T_min_* uncertainties combined in quadrature. Thus,

(4) $$2\sigma \;\left(T_{mean}\right)=1.96\times \sqrt{\left(0.366^{2}+0.135^{2}\right)/2}=\pm 0.382\;{}^{\circ}C$$

This ±0.382 °C represents the field-conditions lower limit of visually-read resolution-limited 2σ uncertainty to be assigned to any global daily mean land-surface meteorological LiG air temperature. During the decade after 1980, transition to MMTS sensors began [65], which is considered below.

In a monthly mean temperature $T_{mean}^{M}=\frac{1}{2n}\overset{n}{\underset{i=1}{\sum }}\left(T_{i}^{min}+T_{i}^{max}\right)$, where *M* is month and *n* is days/month. The uncertainty in *T_mean_* for an average month (30.417 days) is the RMS of the daily means:

(5) 2σ=1.96×(30.417×[0.195230.417=±0.382 °C.

Likewise, for an annual land-surface air-temperature mean:

(6) 2σ=1.96×12×(0.198)212=±0.382 °C.

Noteworthy is that the measurement uncertainty conditioning a temperature anomaly based upon the uncertainty in *T_mean_* alone is, ($T_{mean}^{M}-T_{normal}^{30-year})=T_{anomaly}^{M}$, and $2\sigma_{anomaly}^{M}=1.96\times \pm \sqrt{0.195^{2}+0.195^{2}}=\pm 0.540\;{}^{\circ}C$, where *M* is month.

### 4.3. Sea Surface

The uncertainty in a global average temperature requires a weighted combination of the lower limit uncertainties in land-surface and sea-surface temperatures. Spirit LiG thermometers make no appearance in SSTs. For the uncertainty in a mean of mercury LiG SSTs, only the 2σ = ±0.265 °C resolution and 2σ = ±0.027 °C from Hg LiG non-linearity apply. Thus, the LiG lower limit of laboratory resolution for SSTs is, $2\sigma_{SST}=1.96\times \sqrt{{\left(0.135\right)}^{2}+{\left(0.0138\right)}^{2}}=\pm 0.266\;{}^{\circ}C.$

### 4.4. Global

The global land plus SST uncertainties are scaled by their respective global surface area and combined in quadrature. Thus,

(7) $$2\sigma_{G}=1.96\times \sqrt{0.7\times {\left(0.136\right)}^{2}+0.3\times {\left(0.195\right)}^{2}}=\pm 0.306\;{}^{\circ}C$$

is the lower limit of LiG uncertainty conditioning any global monthly average air temperature compiled prior to 1981.

However, global average air temperature is, typically, presented as an anomaly trend. As previously noted, in taking an anomaly, both the annual mean air temperature and the reference normal, e.g., a 1951–1980 30-year mean, will each be conditioned by the same resolution-limited ±0.308 °C. The 2σ = ±0.308 °C resolution uncertainty will, thus, separately condition both an annual mean and a 30-year normal. In calculating an annual anomaly, uncertainties in the differenced values are added in quadrature [106].

The lowest limit of uncertainty in any global annual LiG-derived air-temperature anomaly prior to 1981 is then found in the combined lower limit of detection, the non-linearity of a LiG thermometer, and the visual repeatability of measurement. The laboratory-standard resolution-limited uncertainty in a global annual air-temperature anomaly is thus

(8) $$\pm 2\sigma_{Ga}=1.96\times \sqrt{{\left(0.156^{2}\right)}_{AM}+{\left(0.156^{2}\right)}_{N}}=\pm 0.432\;{}^{\circ}C$$

where subscript *Ga* is global anomaly, *AM* designates *annual mean* temperature, and *N* designates the 30-year *normal*-period temperature mean. The statistics requiring propagation of both sources of uncertainty into differences indicate that the uncertainty of an anomaly is always greater than the uncertainty in an annual mean or in a 30-year reference normal.

**Figure 17:**
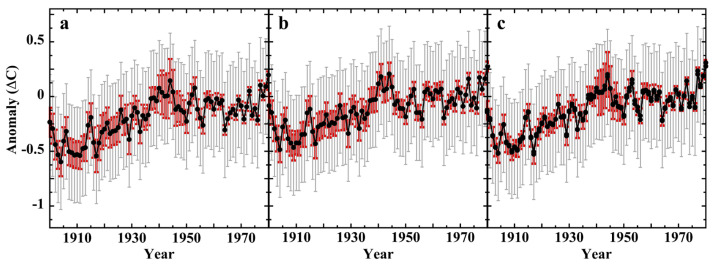
(Points), 1900–1980 global air-temperature anomalies for: (**a**) HadCRUT 5.0.1.0 (published through 2022); (**b**) GISSTEMP v4 (published through 2018); and (**c**) Berkeley Earth (published through 2022). Red whiskers: the published 2σ uncertainties. Grey whiskers: the uniform 2σ = ±0.432 °C uncertainty representing the laboratory lower limit of instrumental resolution for a global average annual anomaly series prior to 1981.

Figure 17 shows the foundational 2σ = ±0.432 °C instrumental uncertainty in a temperature anomaly applied to the HadCRUT 5.0.1.0, the GISSTEMP v. 4, and the Berkeley Earth land–ocean annual temperature anomaly records [11,12,13]. The 19th century anomalies were excluded because the unknown contributions of Joule-drift render the entire early temperature trend unreliable (*cf.* Section 4.7). The plots terminate at 1980 because min–max temperature system (MMTS) sensors began to replace the LiG thermometer in land stations during the following decade.

Nevertheless, across the first 80 years of the 20th century, the analytically basic uncertainty following from the resolution of LiG thermometers alone yields 2σ uncertainty bounds averaging 4× the published 95% confidence interval for the global air-temperature record of 1900, more than 5× after 1950, and 13× by 1980. The uncertainty stemming from the laboratory resolution limit of LiG thermometers alone is sufficient to obscure the rate and magnitude of climate warming since 1900.

### 4.5. Sensor-Transfer Functions

The 1980s saw a change-over from the LiG thermometer in a louvered Stevenson/CRS shield to the new MMTS instrument featuring a thermistor in a gill shield, each of which is naturally ventilated [65,213,214]. Prior to the final change-over, several months of side-by-side CRS/MMTS comparative temperature measurements were carried out. The side-by-side measurement series allowed detection of any measurement bias offset that may distinguish the original sensor from the replacement sensor. The mean bias differentiating the measured temperatures was then removed by adjusting the mean of one temperature series into the mean of the other [65]. This offset correction is the transfer function. Difficulties applying this method to individual station series [214] are not examined here.

Generally, for an existing CRS sensor, *S_1_*, the mean of the temperature series measured during the observational overlap time is *T_m_*_1_ = *T_m_*_0_ + *ε_m_*_1_, where *T_m_*_0_ is the unknown physically correct air-temperature mean and *ε_m_*_1_ is the unknown mean-measurement error. The homologous statement for an entering MMTS sensor, *S*_2_, is *T_m_*_2_ = *T_m_*_0_ *+ ε_m_*_2_. The transfer-function bias correction, *β*_1,2_, to be applied is the difference between the means of the two temperature-measurement series,

(9) $$\beta_{1,2}=T_{m1}-T_{m2\;}=\left(T_{m0}+\varepsilon_{m1}\right)-\left(T_{m0}+\varepsilon_{m2}\right)=\left(T_{m0}-T_{m0}\right)+\left(\varepsilon_{m1}-\varepsilon_{m2}\right)=\Delta \varepsilon_{m1,2}$$

where Δ*ε*_m1,2_ is the difference of the unknown error means. That is, the transfer function, *β_1,2_*, used to correct the mean *S*_1_ → *S*_2_ offset bias is just Δ*ε*_m1,2_—the difference of the respective unknown measurement error means. The mean uncertainty in each measurement of a temperature–time series is $\pm u_{T}=\sqrt{\frac{\sum_{i=1}^{n}{\left(\varepsilon_{i}^{t}\right)}^{2}}{n}}$, where $\varepsilon_{i}^{t}$ is the derived error in the *ith* measured temperature in a field-calibration experiment consisting of *n* measurements (*cf*. Section 3.3).

**Figure 18:**
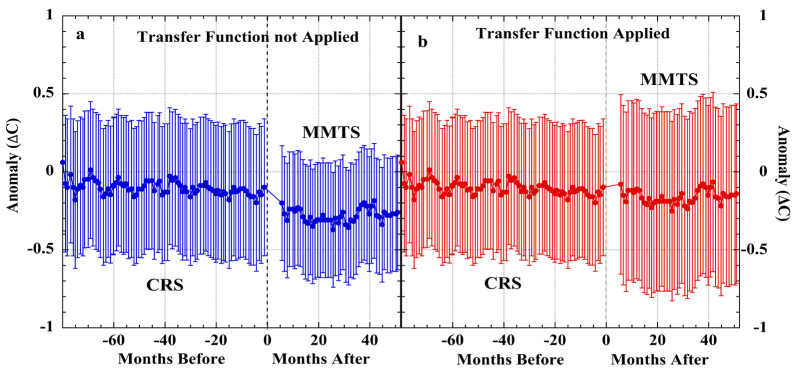
Illustration of the effect of applying a transfer-function adjustment after side-by-side temperature measurements with a LiG thermometer in a cotton region shelter and an MMTS sensor. The points are monthly average air-temperature anomalies from the CRS sensor (left) or the MMTS sensor (right) of Figure 4 from Quayle and associates [65]. The five months following zero were excluded from the original analysis. (**a**) Whiskers are 1σ field-calibration uncertainty bounds for air-temperature measurements from a sensor within the unaspirated CRS (±0.29 °C) or MMTS (±0.28 °C) shield [31]. These increased to ±0.41 °C and ±0.40 °C, respectively, after differencing to the anomaly (*cf.* Section 3.1.1 and Table 6). (**b**) Whiskers are 1σ uncertainty bounds after transfer-function adjustment. The uncertainty of the MMTS anomalies has increased to ±0.57 °C after adjustment because the CRS and MMTS temperature series are no longer independent. Following adjustment, the CRS uncertainty entered the MMTS series and propagated into the MMTS uncertainty as the root–sum–square.

In applying *β*_1,2_ to the *S*_2_ temperature–time series, the two measurement series are no longer independent, because the Δ*ε*_m1,2_ = *β*_1,2_ mean error offset from *S*_1_ enters into every subsequent temperature measured by *S_2_*. Adjusting the mean of *S*_2_ into the mean of *S*_1_ (or vice versa) means the systematic uncertainty, *±u_T_*, of *S*_1_ enters into the new *S*_2_ series. This situation is illustrated in Figure 18, for the published CRS and MMTS adjustment series [65].

By way of explanation, the physically correct temperatures, estimated by the *S_1_* measurements, are unknown. The extent of knowledge is that the correct temperature mean very likely resides somewhere within the *S*_1_ uncertainty range. Following adjustment with a transfer function, *S_2_* engages the uncertainty of the *S*_1_ mean. As the entire *S*_2_ series is offset by Δ*ε*_m1,2_, the *±u_T_* for the *S*_1_ mean propagates into the uncertainty of every *S*_2_ air temperature as the root–sum–squared. The total uncertainty in each *S*_2_ measured temperature then becomes $\pm u_{2}^{adj}=\sqrt{u_{1}^{2}+u_{2}^{2}}$, such that the $\pm u_{2}^{adj}>\pm u_{2}$, i.e., the transfer-function adjustment increases the uncertainty. Thus, transfer functions should cease to be used to adjust temperature series following instrumental changes in surface meteorological stations.

Similar problems will arise on making transfer-function adjustments following sensor-location moves. Field-calibration errors change with physical location because mean environmental variables shift [27], which, in turn, may produce a different *±u_T_* for the identical sensor [40]. Given a physical move, the sensor $\pm u_{T}^{ante}⋚\pm u_{T}^{post}$ but, in any case, if the pre- and post-move temperature series means are adjusted using a transfer function to remove a step, $\pm u_{T}^{ante}$ must propagate into the subsequently measured air temperatures.

### 4.6. A Lower Limit of Uncertainty in the Global Averaged Surface Air Temperature to 2010

The uncertainties due to resolution and to systematic measurement error are now combined to produce a global average anomaly trend conditioned with metrologically valid lower-limit uncertainty bounds. For the years 1900–1980, the instrumental resolution uncertainties in land-surface global air temperatures 2σ = ±0.382 °C and SSTs 2σ = ±0.266 °C, are brought down from Section 4.2 and Section 4.3.

To these must be added the uncertainty stemming from the non-random systematic measurement error revealed by the calibration experiments of land-surface air-temperature sensors. The several CRS and MMTS calibrations from Table 6 are assumed to have equivalent statistical validity and to adequately sample the impacts of varying physical environments. The unweighted mean of known LiG/CRS systematic calibration error is 2σ = ±0.58 °C. Following 1990, the lower-limit calculation allows MMTS sensors to have replaced LiG/CRS sensors worldwide. The mean of MMTS calibration uncertainty 2σ = ±0.56 °C is, therefore, applied under the same assumptions. Following 2005, the uncertainty in MMTS in land-surface temperatures is replaced by the 2σ = ±0.1 °C resolution and 2σ = ±0.47 °C electronic uncertainty (over −20 °C to 30 °C) in the Climate Research Network (CRN) sensor [212,215].

For SSTs, the available bucket and engine-intake field calibrations show that shipboard SST measurement errors are, likewise, not random. The uncertainties attached to bucket SSTs, 2σ = ±0.4 °C and engine-intake SSTs 2σ = ±2 °C are taken from the calibration experiments reported by Tabata carried out aboard the *C.F.A.V. Endeavor* oceanographic vessel [81]. The calibration-error difference, *ε_EI_* − *ε_B_* = *±*0.8 °C, is well within the set of reported mean-measurement differences described above.

A small but significant fraction of SSTs falls under unknown methods of measurement [176]. For these, the lower-limit calculation required assignment of the bucket uncertainty prior to 1931, followed by zero from 1932 to 1945. After 1946, uncertainty of unknown methods was calculated as the RMS of 0.33 bucket, 0.33 engine-intake, and 0.33 bathythermograph (BT) measurements (2σ_bathy_ = ±0.3 °C) [216]. The resulting systematic SST uncertainty 2σ = ±0.62 °C is dominated by engine-intake uncertainty. One calibration of surface drifters showed only random temperature errors [217]. However, buoy temperature-measurement errors may not be random everywhere [86,89].

Fractions of SSTs entering the record from bucket, engine-intake, and unknown methods were derived from published data [176]. All final uncertainties in air temperature are root–sum–squares of entering calibration errors or uncertainties. The uncertainty in global annual temperature from a land-surface LiG thermometer is,

(10) $$\pm u_{LS}=\sqrt{u_{res}^{2}+u_{acc}^{2}+u_{nonlin}^{2}+u_{sys}^{2}},$$

where subscript *res* is visual resolution, *acc* is accuracy, *nonlin* is non-linearity of response, and *sys* is the mean systematic measurement error induced by environmental variables. For MMTS sensors, there was no term for visual resolution or non-linearity. The uncertainty in each annual LiG SST is,

(11) $$\pm u_{SST}=\sqrt{{\left(f_{b}\times \varepsilon_{b}\right)}^{2}+{\left(f_{EI}\times \varepsilon_{EI}\right)}^{2}+{\left(f_{unk}\times \varepsilon_{unk}\right)}^{2}},$$

where *f* is fraction of SSTs by that method, as provided in published work [176]. Subscript *ε* is error of the method, *b* is bucket, *EI* is engine-intake, and *unk* is unknown method. The sum of fractions, *f_b_ + f_EI_ + f_unk_* = 1. The fractional contribution of each method to SST and their derivation are provided in the tab-delimited text file, “Calculation of SST Fractions” in the Supplementary Materials. The final annual uncertainty in global air temperature was calculated as,

(12) $$\pm u_{G}=\sqrt{0.3\times u_{LST}^{2}+0.7\times u_{SST}^{2}},$$

where subscript LST is land-surface air temperature and SST is sea-surface temperature. Table 8 provides the separate uncertainties entering the global record.

**Figure 19:**
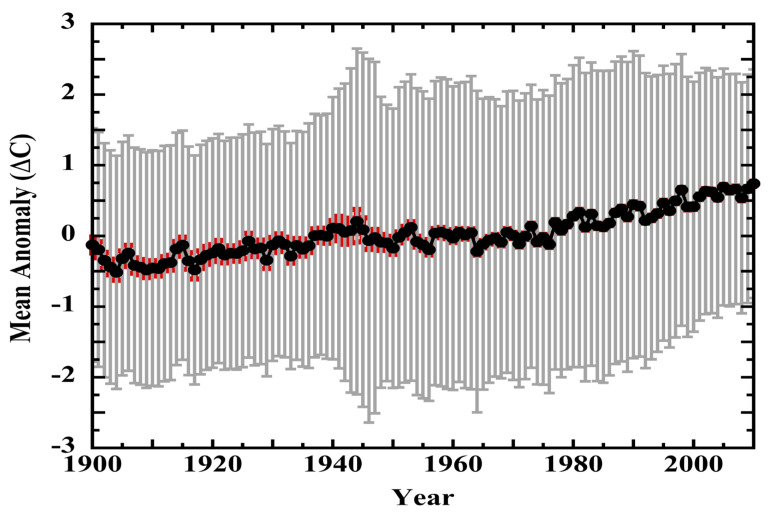
(Points), the mean of air-temperature anomalies published by the UK Met Office Hadley Centre/Climatic Research Unit, the Goddard Institute for Space Studies, and Berkeley Earth [11,13,218]. Each anomaly series was adjusted to a uniform 1951–1980 normal prior to averaging. The 19th century anomalies were excluded because of the sure contamination with Joule-drift. (Red whiskers), the 2σ RMS of the combined published uncertainties of the three anomaly records. (Grey whiskers), the 2σ uncertainty in the anomaly mean, stemming from the lower limit of laboratory resolution and the calibration mean of systematic error due to environmental variables, combined in quadrature. See the text for details. The details of the calculation may be found in the tab-delimited ASCII-column text files in the Supplementary Materials.

As before, in calculating the uncertainty in an anomaly, the uncertainty in air temperature must be combined in quadrature with the uncertainty in a 30-year normal (Table 9). The globally averaged surface air-temperature-anomaly record amended with the ±2σ (95%) lower limit of uncertainty resulting from these calculations is shown in Figure 19. In constructing Figure 19, the 1951–1980 NASA/GISS 30-year normal was chosen [19], with RMS uncertainty 1σ = ±0.758 °C (*cf.* Table 9). The details of the calculation may be found in the tab-delimited ASCII-column text files in the Supplementary Materials. The uncertainty bounds represent a lower limit, including:

- The accuracy—the limit of detection of high-quality 1 °C/division mercury LiG thermometers;
- The resolution—the limit of visual repeatability of a temperature reading under ideal laboratory conditions;
- The non-linearity of LiG response to temperature;
- The land-station systematic field-measurement uncertainty from calibrations of well-sited and well-maintained sensors;
- The SST bucket, engine-intake, and bathythermograph uncertainties from calibrations by trained personnel aboard an ocean research vessel.

The majority of uncertainty after 1945 derives from inclusion of engine-intake measurements with their calibration uncertainty. Over 1981–1989, CRS error was transitioned to MMTS error by linear interpolation. LiG resolution was not included after 1989, while only MMTS errors were included for land-surface error from 1990–2004. After 2005 for land-surface air temperatures, only the measurement uncertainty of the Climate Research Network sensor was included. SST uncertainties included LiG resolution and calibration uncertainty. Random errors from drifting and moored buoys were not included in the total uncertainty. Anomalies prior to 1900 were excluded because Joule-drift renders the early record unreliable through 1890 at least. Uncertainty increases after 1945 because engine-intake SSTs come to dominate the record in the second half of the 20th century. Table 9 summarizes the temperature anomaly statistics for the nine 30-year normal periods between 1901 and 2010.From Figure 19, the mean global air-temperature-record anomaly over the 20th century (1900–1999) is 0.74 ± 1.94 °C. The 2σ = ±1.94 °C uncertainty does not indicate a range of possible temperatures but, rather, the range of ignorance over which no information is available [219,220,221]. That is, the physically correct mean anomaly may be anywhere within that range. Relative to the 1951–1980 normal, the anomaly mean ± 2σ RMS uncertainty for 1900–1945 is −0.21 ± 1.7 °C, for 1946–1980, −0.01 ± 2.1 °C, for 1981–2004, 0.37 ± 2.0 °C, and for 2005–2010, 0.66 ± 1.6 °C. Changing the normal period to the originally proposed 1901–1930 normal [1,222], reduces the anomaly uncertainty by about 1/3. Reporting the air-temperature trend rather than the anomaly trend would reduce the mean uncertainty by about two-fold (1951–1980 normal).

### 4.7. Joule-Drift

Joule-drift is discussed here because it does not enter into appraisals of the 20th century anomaly record. However, the Joule-drift of lead-glass or soft-glass thermometers fatally compromises temperature measurements prior to 1890. On the continuous use of such thermometers, Joule-drift would have added a spurious warming trend of about 0.6–0.7 °C/°F per century to a surface-station temperature record through the 19th century. This problem was well-known to contemporaneous meteorologists. Thu*s*, “*In considering the well-worn question of the zero-movements of thermometers, … for example* [*regarding*] *the thermometer … verified at Kew Observatory … I knew that the zero would probably rise and that the amount of the rise would not be the same in my case as in that of others and that therefore, I must obtain the index-error experimentally”* [129]. Similarly, “*The upward displacement of the zero in mercury thermometers used in the Specola in the second half of the 19th century was about 0.3–0.6 °C, as demonstrated by the accurate measurements carried out in the second half of the 19th century, noted in the observation registers*” [112]. Specola Astronomic Observatory is now the Astronomical Observatory of Padua. However, the previously well-understood lesson of Joule-drift has evidently been latterly forgotten.

Recovery of early historical air-temperature series is an on-going major project [223,224,225,226,227]. However, quality-assurance methodologies for early temperature series do not mention instrumental detection limits or correction for LiG non-linearity, and stand silent on the impact of Joule-drift [223,225,228,229]. Individual reports of recovery of early historical land-surface air-temperature series, likewise, do not mention the problems of LiG thermometer non-linearity or of Joule-drift [230,231,232,233,234,235,236,237,238,239,240,241,242], with exceptions as notable as they are rare [112,113,115,243,244,245,246]. The unknown but inevitable impact of systematic measurement errors that accrue to naturally ventilated sensors is, likewise, by-passed in silence. Incorporation of these uncorrected and unreliable early historical air-temperature series into modern compilations will necessarily produce spurious trends and unfounded conclusions.

The X-ray-emission analysis reported here demonstrates that some meteorological thermometers continued to be constructed of lead-glass until at least 1900. No recognition of Joule-drift, detection limits, or non-linearity of response appears in the modern compilations of meteorological station air temperatures [83,247,248]. Likewise, temperature corrections for Joule-drift or nonlinearity are not mentioned in the mid-20th century initializing reports of hemispheric or global average air temperatures, where the fundamentals of methodology should appear [3,19,51,52,53,83,249,250]. Nor are they found at the contemporary GISSTEMP explanatory website [251], nor the reported Berkeley Earth global temperature record [252].

Undetected Joule-drift in the 19th century global air-temperature record renders uncertain any warming trend prior to 1900. Correction for the likely impact of Joule-drift prior to 1900 appears impossible.

## 5. Conclusions

This work has presented an analysis that is some 40 years overdue, namely, critical application of metrological standards of instrumental resolution, calibration, measurement error, and uncertainty [253,254,255], to meteorological air-temperature instruments and measurements.

### 5.1. Major Findings

With respect to the published global air-temperature record, the major findings are:

- The accuracy limit of LiG meteorological thermometers, 2σ = ±0.11 °C/°F, had been ignored;
- The laboratory lower-limit ideal of visual repeatability of LiG thermometer, 2σ = ±0.144 °C/°F, had been ignored;
- The published uncertainty of the 1900–1980 global average air-temperature anomaly record was less than the combined 2σ = ±0.432 °C laboratory ideal lower limit of resolution of high-quality LiG thermometers;
- Joule-drift of pre-1890 lead-glass or soft-glass thermometers had been ignored, but renders unreliable the early air-temperature record through the 19th century;
- Lead-glass meteorological thermometers were still manufactured and entering use in 1900;
- Land- and sea-surface temperatures had not been corrected for the non-linear response of LiG thermometers;
- Systematic measurement error produced by naturally ventilated land-surface air-temperature sensors is not random;
- Systematic land-surface air-temperature-measurement error is correlated across sensors;
- The semivariogram method does not reveal mean SST measurement error, but rather, half the mean difference in error, i.e., 0.5Δε_µ_;
- The mean error in SST measurements remains unknown (as does the marine wind measurement error mean);
- Bucket SST measurement error is typically not random;
- Engine-intake SST measurement error is not random;
- The distribution of ship SST measurement error varies with each trip, with the crew (and even with the watch), and between ships;
- Means of ship SST error distributions are themselves not randomly distributed;
- Turbulence caused by the ship (platform) itself generally obviates the correspondence of the measurement to the undisturbed state of surface waters. *In-situ* SST measurements that may be accurate, will nevertheless be physically incorrect.

LiG thermometer Joule-drift has rendered the entire early air-temperature record through the 19th century unreliable. Field-calibration experiments of air temperature and ship SST sensors uniformly disconfirm the assumption that air temperature and SST measurement error is strictly random. Exceptions may include bucket SST measurements carried out by methodologically trained personnel and SSTs measured using modern buoys.

The compilation of land- and sea-surface LiG uncertainty yield a 1900–2010 global air-temperature record anomaly of 0.86 ± 1.92 °C (2σ), which renders impossible any conclusion regarding the rate or magnitude of climate warming since 1850 or earlier.

### 5.2. Involve the ASPE

Before any further policy decisions are to be made on the grounds of a warming climate, full and complete analysis of the air and sea-surface temperature record must be engaged, completed, and reported by multiple independent professionally disinterested and metrologically expert third-party engineering groups. Only this will resolve the forefront problem of reliability brought into focus here. Notions of unprecedented warming and disastrous outcomes deriving from the previously published air-temperature record are scientifically unsustainable. Their wider significance must, instead, await unconflicted metrological and economic analyses. Causality remains deeply at issue [256,257,258,259,260,261].

A first order of business might be to fully evaluate the systematic field-measurement errors of surface air-temperature meteorological field stations. Climate Research Network aspirated sensors, modified to produce more highly accurate measurements [212], might be placed near selected CRS and MMTS field-station sensors of the Global Historical Climatology Network. Automation to retrieve LiG thermometer readings is available [262,263]. The systematic measurement errors made by CRS/LiG and MMTS sensors in working meteorological field-stations could then be evaluated under a complete sampling of the impacts of environmental circumstance. A full inventory of poorly-sited surface stations should be included in the experiment so as to ensure a comprehensive survey [264,265,266,267,268,269].

These calibration experiments would provide the first thorough evaluation of the reliability of land-surface field station air-temperature measurements. The integrity of individual, regional, and, ultimately, global land-surface air temperatures would become available. The calibration uncertainties derived would be applicable to a historically valid estimate of uncertainty in the annually resolved global average land-surface air-temperature record dating back to 1900. The presence and magnitudes of inter-sensor cross-correlations across increasing distances could then also be determined. The described calibration experiment would be expensive. However, the cost is trivial compared to the ~39 billion USD spent on scientific and technical research between 1990–2018 [270], to ameliorate a problem now known to be objectively invisible (this work, and [256,257,258,271,272]).

The global averaged surface air-temperature record has been central to notions of unprecedented and dangerous climate warming for at least 35 years [19,20,21]. The 1990 Summary for Policymakers of the First Assessment Report (1AR) produced by the Intergovernmental Panel on Climate Change (IPCC) warned of, “*a rate of increase of global mean temperature during the next century of about 0.3 °C per decade …, this is greater than that seen over the past 10,000 years* [22]”. Likewise, the 2021 IPCC 6AR Summary for Policymakers begins, “*It is unequivocal that human influence has warmed the atmosphere, ocean and land* [24]”. However, this and prior work show that neither statement can be sustained on scientific grounds [7,69,256,257,258,259,260,261,271].

Given the scope of the warnings and the enormous expenditures and economic dislocations in the name of climate warming [273], it is not an exaggeration to suppose that a comprehensive societal effort would have been expended to ensure the scientific basis validating a cause for worry. However, a comprehensive competent third-party metrological evaluation of the historical air-temperature record by precision engineers is notably absent. At the same time, the present work has shown that production of the record has sorely lacked the necessary attention to detail. Critical assumptions remained untested, and analyses wanted the deep care demanded by scientific rigor. The first order of business in experiment is to evaluate the reliability of the instrument, followed by assessing the quality of the data. Remarkably, the current global air-temperature record evidences no understanding of LiG thermometers, of their history, or of their metrology.

Very evidently, a professionally competent and disinterested third party must be commissioned to produce a full and rigorous instrumental engineering evaluation of the historical temperature record. It is here recommended that the American Society for Precision Engineering constitutes one such independent and competent third party. Along with precision engineering societies from other countries, their full, independently replicated, and delivered evaluations of meteorological air temperatures must precede any further actions.

### 5.3. Final Conclusions

Direct evidence of a warming climate since the 19th century includes the lengthened growing season, the revegetation of the far North, and the poleward migration of the northern tree line [274,275,276,277,278,279,280,281,282,283]. However, at the 95% level of uncertainty, neither the rate nor the magnitude of 19th or 20th century warming can be known. A more detailed appraisal of errors may modify the uncertainty bounds, but an alternative conclusion is unlikely.

The 20th century surface air-temperature anomaly, 0.74 ± 1.94 °C (2σ), does not convey any knowledge of rate or magnitude of change in the thermal state of the troposphere. Climate alarm on that account is unjustifiable. The Joule-drift that certainly plagued all LiG thermometers manufactured prior to 1885 obviates the reliability of earlier air-temperature measurements. The global averaged surface air-temperature anomaly record cannot sustain any notion of unprecedented climate warming over the last 200 years, or over any other timespan.

## Figures and Tables

**Table 1:** NIST 1 °C/division Mercury LiG Thermometer Calibration Resolution Limits (±2σ, °C).

	Eye Alone	Magnifying Lens
accuracy limit (resolution) ^a^	0.300	0.114
visual repeatability	0.144	0.144
per-measurement uncertainty ^b^	0.326	0.178
anomaly uncertainty ^c^	0.461	0.252

^a^ Three significant figures are used throughout to reduce round-off error. ^b^ root–sum–square of resolution and visual repeatability. ^c^ Uncertainty in an anomaly is the root–sum–square of the uncertainties in the differenced magnitudes.

**Table 3:** Fit Parameters for Stevenson Shield Calibration Error.

	Gaussian 1 (*f_m_*, σ)	Gaussian 2 (*f_m_*, σ)	Lorentzian (*f_m_*, Γ)	Fit r^2^
Stev. (Wood)	0.108, 0.230	0.024, 0.096	2 × 10^−4^, 0.084	0.999
Stev. (PVC)	0.041, 0.172	8.3 × 10^−3^, 0.052	−0.029, 0.036	0.943

**Table 4:** KNMI Average Correlations of Test-Screen Temperature Error 1989–1995.

	Socrima	Young Gill	Stv. PVC	Stv. Wood	Stv. PVC asp	Vaisala	Young asp II	KNMI asp
Socrima	1	0.28	---	0.64	0.14	0.18	0.15	0.30
Young Gill		1	0.33	0.28	0.27	0.54	0.32	0.60
Stv. PVC			1	0.88	---	0.30	---	0.07
Stv. Wood				1	0.04	0.18	0.06	0.17
Stv. PVC asp					1	0.28	0.47	0.36
Vaisala						1	0.44	0.76
Young asp II							1	0.35
KNMI asp								1

Average correlation is the RMS positive root. Some screens had no measurement-overlap periods. All screens were naturally ventilated, except those marked ‘asp’.

**Table 5:** Measurement Error in Naturally Ventilated Fine-Wire Thermocouple Sensors.

Aspirated Ref. →	Thermocouple Yankee 2010		PRT MetOne 327-C	
Test shield ↓	Error (µ ± σ; °C)	Shapiro–Wilk	Error (µ ± σ; °C)	Shapiro–Wilk
Gill (N = 2072)	0.11 ± 0.34	0.696, *p* < 0.001	0.12 ± 0.45	0.968, *p* < 0.001
Custom *(N = 691*)	0.11 ± 0.40	0.701, *p* < 0.001	0.12 ± 0.49	0.945, *p* < 0.001

Data are from the Savannah River National Laboratory calibration experiment [171].

**Table 6:** RMS Calibration Measurement Uncertainty within Naturally Ventilated Shields.

Sensor Shield	Uncertainty (±°C)	Calib. Sensor	Reference
Stv. Wood ^a^	0.20	asp. PRT ^b^	[168]
Stv. PVC ^a^	0.19	asp. PRT	[168]
HOBO (25 avg) ^c^	0.43	asp. PRT	[90]
CRS ^d^	0.53	asp. PRT	[31]
MMTS ^e^	0.25	asp. PRT	[31]
MMTS ^f^	0.28	asp. thermistor	[32]
Gill ^g^	0.26	asp. PRT	[31]
Gill ^h^	0.45	asp. PRT	[171]
Custom Plate ^h^	0.49	asp. PRT	[171]
Gill ^h^	0.36	asp. Therm. ^i^	[171]
Custom Plate ^h^	0.40	asp. Therm. ^i^	[171]
Thermocouple ^k^	2.20	Sonic Anem. ^j^	[36]
R. M. Young ^k^	2.95	Sonic Anem. ^j^	[36]
MMTS ^k,l^	0.31	asp. CRN ^m^	[172]
Stv. Wood (lg)	0.24	asp. PRT	[68]
Stv. Wood (sm)	0.23	asp. PRT	[68]

^a^ Wooden or polyvinylchloride Stevenson screen. ^b^ aspirated platinum resistance thermometer. ^c^ Average of 25 naturally ventilated shields. ^d^ Cotton region shelter. ^e^ Min–max temperature system. ^f^ over a snow-covered surface. ^g^ PRT, Gill plate shield. ^h^ thermocouple. ^i^ aspirated thermocouple wire. ^j^ sonic anemometer. ^k^ Over a snow-covered surface at 2700 m on Alpine Plaine Morte Glacier, Switzerland. ^l^ December–February day–night average error scaled to reflect 10 daylight hours at Lincoln, NB, USA (40°48′00″ N; 96°40′00.012″ W). ^m^ Standard Climate Research Network aspirated shield.

**Table 7:** Estimate of Uncertainty in Global Averaged Temperature from LiG Non-Linearity.

Mercury	Spirit
$Var_{C}=0.5\times {\left(0.017\;{}^{\circ}C\right)}^{2}+0.5\times {\left(0.556\times 0.017\;{}^{\circ}F\right)}^{2}$	$Var_{C}=0.5\times {\left(0.33\;{}^{\circ}C\right)}^{2}+0.5\times {\left(0.556\times 0.33\;{}^{\circ}F\right)}^{2}$
$Uncertainty\;Variance=0.00019\;{}^{\circ}C^{2}$	$Uncertainty\;Variance=0.0713\;{}^{\circ}C^{2}$
1σ_non-linearity_ = ±0.0138 °C	1σ_non-linearity_ = ±0.267 °C

*Var_C_* is the variance in Celsius-squared.

**Table 8:** Lower Limit of Uncertainty (±2σ) Entering the Global Air-Temperature Record.

Land Surface		Sea Surface	
Instrumental		Instrumental (LiG; 1 °C/division) ^a^	
accuracy (LiG; 1 °C/division) ^a^	0.30	accuracy	0.30
visual repeatability (LiG; 1 °C/division) ^a^	0.144	visual repeatability	0.144
non-linearity (LiG; 1 °C/division) ^a^	0.371	non-linearity	0.017
MMTS ^b^	0.196		
Systematic		Systematic	
Stevenson/CRS ^b^	0.58	bucket	0.30 ^d^
MMTS ^b^	0.56	engine-intake	2.0 ^d^
Instrumental (USCRN) ^c^		bathythermograph	0.30 ^e^
sensor resolution	0.10		
self-heating ^f^	0.48		

^a^Table 1. ^b^ Table 6. ^c^ ref. [212]. ^d^ ref. [81]. ^e^ ref. [216]. ^f^ ref. [212].

**Table 9:** 30-Year Normal Periods, Anomalies, Trends, and Uncertainties.

Normal Period	Anomaly Mean (Δ°C) ^a^	RMS 2σ Uncertainty (±°C)	100-Year Trend (Δ°C) ^a^
1901–1930	−0.30	0.71	0.79
1911–1940	−0.20	0.73	1.23
1921–1950	−0.09	1.15	0.74
1931–1960	−0.03	1.41	0.26
1941–1970	−0.91	1.57	−0.31
1951–1980	0.0	1.48	0.40
1961–1990	0.09	1.49	1.50
1971–2000	0.24	1.44	1.82
1981–2010	0.43	1.26	0.0

^a^ Relative to a 1951–1980 normal.

## Data Availability

All data supporting the reported results can be found at the cited sources and in the Supplementary Materials documents.

## Supplementary Materials

The following Supplementary Materials can be downloaded at: https://www.mdpi.com/article/10.3390/s23135976/s1, Figure S1: Field measurement-error of naturally ventilated PRT sensors; Table S1 KNMI Calibration Error Correlations for calibration year 1989; Table S2 KNMI Calibration Error Correlations for calibration year 1990; Table S3 KNMI Calibration Error Correlations for calibration year 1991; Table S4: KNMI Calibration Error Correlations for calibration year 1992; Table S5: KNMI Calibration Error Correlations for calibration year 1993; Table S6: KNMI Calibration Error Correlations for calibration year 1994; Table S7: KNMI Calibration Error Correlations for calibration year 1995; Figure S2. Error of HOBO #1 through HOBO #25 air temperature sensors; Table S8: HOBO Field Measurement analysis; Figure S3: Histogram of HOBO error means; Figure S4. Combined systematic measurement error from 25 HOBO sensors; Figure S5: digitized buoy air temperature measurements; Figure S6: Correlation of buoy air temperature measurement errors; Table S9: Buoy Sensor Error Correlation Matrix; Figure S7: Buoy air temperature measurement errors; Figure S8: Combined buoy air temperature measurement error; Table S10: Fitting Parameters for Buoy Sensor Measurement Error; Figure S9: Stevenson screen calibration and correlation of custom errors; Figure S10: Measurement error: PRT/MetSpec “large” plastic Stevenson screen; Figure S11: Fits to differenced bucket SST measurements; Figure S12: Difference of SSTs, WMO global survey; Figure S13: Frequency of (*T_b_* − *T_E_*), 25–49.9° N&S, all wind speeds; Tab-delimited file: Calculation of Global SST&Land Uncertainty.txt; Tab-delimited file: Calculation of Scaled Fractions & Uncertainties.txt; Tab-delimited file: Calculation of SST Fractions.txt; Tab-delimited file: Uncertainty in Global Air Temperature.txt. Ref. [284] is cited in the Supplementary Materials

## Abbreviations

AGR	radar picket ship
ARGO	Array for Real-Time Geostrophic Oceanography
ASCII	American Standard Code for Information Interchange
BT	bathythermograph
CI	confidence interval
CRN	Climate Research Network
CRS	cotton region shelter
CTD	conductivity–temperature–depth
DER	destroyer escort
FWHM	full width at half maximum
GHCN	Global Historical Climatology Network
GISSTemp	Goddard Institute of Space Studies anomaly record
GSATA	global surface air-temperature anomaly
HadCRUT	UK Met Hadley Centre and University of East Anglia Climate Research Unit anomaly record.
ICOADS	International Comprehensive Ocean–Atmosphere Data Set
IPCC	Intergovernmental Panel on Climate Change
keV	kilo electron-Volt
KNMI	Koninklijk Nederlands Meteorologisch Instituut
LiG	liquid-in-glass
MAE	mixed alkali effect
MMTS	min–max temperature system
MSTS	Military Ship Transport Service
NBS	National Bureau of Standards
NCAR	National Center for Atmospheric Research
NIST	National Institute of Standards and Technology
NMAH	National Museum of American History
PRT	platinum resistance thermometer
PVC	polyvinyl chloride
RMS	root–mean–square
SEA	Sea Education Association
SST	sea-surface temperature
STD	salinity–temperature–depth
USHCN	United States Historical Climatology Network
VOS	voluntary observing ships
WMO	World Meteorological Organization
XRF	X-ray fluorescence
