# Supplementary material for: LiG Metrology, Correlated Error, and the Integrity of the Global Surface Air-Temperature Record
Source: Sensors (Basel). 2023 Jun 27;23(13):5976. doi: 10.3390/s23135976 (PMC10346593; doi:10.3390/s23135976)
Supplement: Supplementary file 1 [file sensors-23-05976-s001.zip › Supplemental Materials Sensors 2435246/Supplemental Material Sensors 2435246.pdf]

Supplementary Material for *LiG Metrology, Correlated Error, and the Accuracy of the Global Surface Air Temperature Record* (2023) MDPI Sensors 2435246

by

Patrick Frank

Table of Contents Page

1. Multi-shield Field Calibration Study, De Bilt 2
Figure S1: Field measurement-error of naturally ventilated PRT sensors 2
Table S1 KNMI Calibration Error Correlations for calibration year 1989 3
Table S2 KNMI Calibration Error Correlations for calibration year 1990 3
Table S3 KNMI Calibration Error Correlations for calibration year 1991 3
Table S4: KNMI Calibration Error Correlations for calibration year 1992 4
Table S5: KNMI Calibration Error Correlations for calibration year 1993 4
Table S6: KNMI Calibration Error Correlations for calibration year 1994 4
Table S7: KNMI Calibration Error Correlations for calibration year 1995 5
II. HOBO shield Field Calibration Study, Ottawa 5
Figure S2. Error of HOBO #1 through HOBO #25 air temperature sensors 5
Table S8: HOBO Field Measurement analysis 6
Figure S3: Histogram of HOBO error means 7
Figure S4. Combined systematic measurement error from 25 HOBO sensors 7
III. Buoy-deployed Shield Calibration Study, Arabian Sea 8
Figure S5: digitized buoy air temperature measurements 8
Figure S6: Correlation of buoy air temperature measurement errors 9
Table S9: Buoy Sensor Error Correlation Matrix 9
Figure S7: Buoy air temperature measurement errors 10
Figure S8: Combined buoy air temperature measurement error 11
Table S10: Fitting Parameters for Buoy Sensor Measurement Error 11
IV. Stevenson screen on a sunny day, steady wind. 11
Figure S9: Stevenson screen calibration and correlation of custom errors 12
V. Calibration: PRT Double-Louvered Modified Stevenson Screen 13
Figure S10: Measurement error: PRT/MetSpec "large" plastic Stevenson screen 13
VI. Bucket minus Bucket distributions 13
Figure S11: Fits to differenced bucket SST measurements 14
VII. WMO Bucket and Engine Intake Temperature Survey 14
Figure S12: Difference of SSTs, WMO global survey 14
VIII Walden 1966, 25-49.9° N, S (T_b-T_E) 15
Figure S13: Frequency of (T_b-T_E), 25-49.9° N&S, all wind-speeds 15
IX References 16

1. Multi-shield Field Calibration Study, De Bilt:

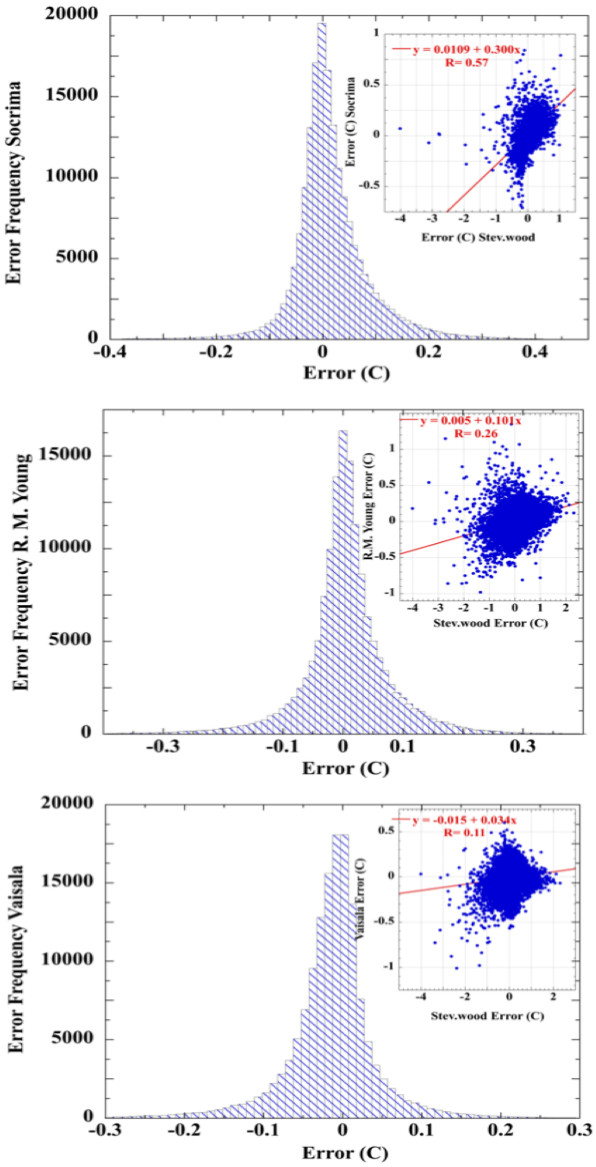

Figure S1: Frequency of (test shield minus $KNMI_{ref}$) field measurement-error of PRT temperature sensors within a naturally ventilated shield: (top) Socrima (N = 179560); (middle), R. M. Young (N = 146450), and; (bottom), Vaisala (N = 146440). Insets: correlation of temperature measurement error with measurement errors produced within a wooden Stevenson screen, over the same time period. Full correlation matrices are provided in Table S1 through Table S7. The calibration experiments were carried out at De Bilt, Netherlands [1].

Table S1: KNMI Calibration Error Correlations for calibration year 1989

Shield	Socrima	Young Gill	Stv pvc	Stv Wood	Stv pvc asp	Vaisala	Young asp II	KNMI.asp
Socrima	---	---	---	---	---	---	---	---
Young Gill		1	0.35	0.38	---	0.57	---	---
Stv pvc			1	0.92	---	0.24	---	---
Stv Wood				1	---	0.26	---	---
Stv pvc asp				---	---	---	---	---
Vaisala						1	---	---
Young Asp II							---	---
KNMI.asp								

Table S2: KNMI Calibration Error Correlations for calibration year 1990

Shield	Socrima	Young Gill	Stv pvc	Stv Wood	Stv. pvc asp	Vaisala	Young asp II	KNMI.asp
Socrima	---	---	---	---	---	---	---	
Young Gill		1	0.30	0.41	---	0.59	---	
Stv pvc			1	0.84	---	0.35	---	-0.02
Stv Wood				1	---	-0.049	---	
Stv pvc asp					---	---	---	
Vaisala						---	---	
Young Asp II							---	
KNMI.asp								1

Table S3: KNMI Calibration Error Correlations for calibration year 1991

Shield	Socrima	Young Gill	Stv pvc	Stv Wood	Stv. pvc asp	Vaisala	Young asp II	KNMI.asp
Socrima		---	---	---	0.22	---	---	0.42
Young Gill		---	---	---	---	---	---	
Stv pvc				---	---	---	---	0.09
Stv Wood					---	---	---	
Stv pvc asp						---	---	0.36
Vaisala						---	---	
Young Asp II							---	
KMNI.asp								

Table S4: KNMI Calibration Error Correlations for calibration year 1992

Shield	Socrima	Young Gill	Stv pvc	Stv Wood	Stv. pvc asp	Vaisala	Young asp II	KNMI.asp
Socrima	1	0.23	---	0.55	0.003	-0.26	-0.097	-0.14
Young Gill		1	---	-0.004	0.12	0.34	0.30	0.52
Stv pvc				---	---	---	----	---
Stv Wood				1	0.044	-0.068	0.030	-0.11
Stv pvc asp					1	0.24	0.45	0.22
Vaisala						1	0.55	0.71
Young Asp II							1	0.40
KNMI.asp								1

Table S5: KNMI Calibration Error Correlations for calibration year 1993

Shield	Socrima	Young Gill	Stv pvc	Stv Wood	Stv. pvc asp	Vaisala	Young asp II	KNMI.asp
Socrima	1	0.33	---	0.71	-0.095	-0.018	-0.20	-0.02
Young Gill		1	---	0.063	0.36	0.61	0.33	0.67
Stv pvc				---	---	---	---	---
Stv Wood				1	---	-0.23	0.085	-0.22
Stv pvc asp					1	0.32	0.48	0.34
Vaisala						1	0.29	0.80
Young Asp II							1	0.28
KNMI.asp								1

Table S6: KNMI Calibration Error Correlations for calibration year 1994

Shield	Socrima	Young Gill	Stv pvc	Stv Wood	Stv. pvc asp	Vaisala	Young asp II	KNMI.asp
Socrima	1	---	---	---	0.0094	---	-0.044	---
Young Gill			---	---	---	---	---	----
Stv pvc				---	---	---	---	--
Stv Wood					---	---	---	---
Stv pvc asp						---	0.49	---
Vaisala							---	---
Young Asp II							1	---
T.KNMI.asp								---˙

Table S7: KNMI Calibration Error Correlations for calibration year 1995

Shield	Socrima	Young Gill	Stv pvc	Stv Wood	Stv. pvc asp	Vaisala	Young asp II	KNMI.asp
Socrima	1	---	---	---	0.19	---	0.19	---
Young Gill			---	---	---	---	---	---
Stv pvc				---	---	---	---	---
Stv Wood					---	---		---
Stv pvc asp					1	---	0.47	---
Vaisala							---	---
Young Asp II							1	---
KNMI.asp								

II. HOBO shield Field Calibration Study, Ottawa

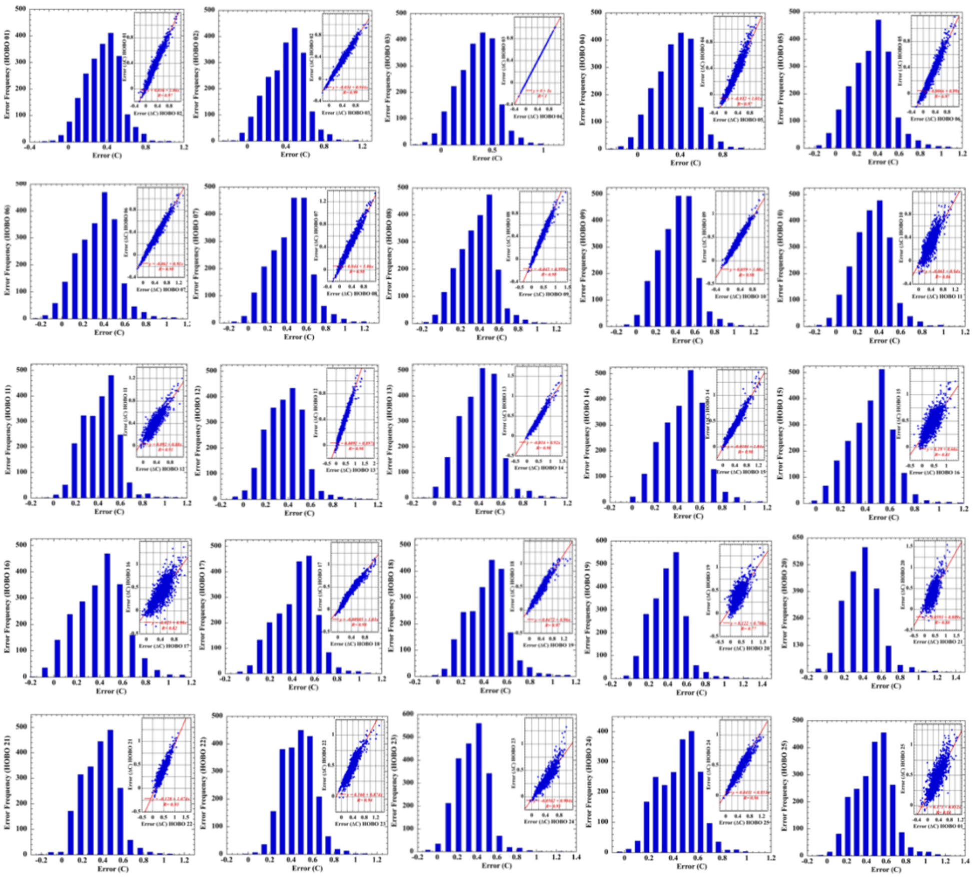

Figure S2. Histogram of frequency of error of HOBO #1 through HOBO #25 air temperature sensors consisting of a thermistor housed in a HOBO shield [2]. The reference was a MetOne 063 high precision thermistor housed in a 076B aspirated radiation shield. Insets: Pair-wise HOBO measurement error correlation plots stepping through the 25 sensors (HOBO #1 vs HOBO #2; HOBO #2 vs HOBO #3; etc.). The final correlation plot is HOBO #25 vs HOBO #1). See text for details.

Table S8: HOBO Field Measurements: Uncertainty[a], Lag-1 Auto-correlation R, and Shapiro-Wilk Test for Normality of the Error Histogram

HOBO#	RMS Error (μ±σ, C)	Correlation R	S-W(2160)[b]	p-value	Random Error
1	0.35±0.40	0.97	0.996	<0.001	No
2	0.40±0.45	0.99	0.992	<0.001	No
3	0.35±0.39	1.00	0.994	<0.001	No
4	0.35±0.39	0.97	0.994	<0.001	No
5	0.34±0.39	0.97	0.988	<0.001	No
6	0.33±0.39	0.98	0.990	<0.001	No
7	0.43±0.48	0.98	0.988	<0.001	No
8	0.37±0.41	0.98	0.991	<0.001	No
9	0.41±0.45	0.98	0.986	<0.001	No
10	0.33±0.37	0.86	0.992	<0.001	No
11	0.42±0.44	0.91	0.985	<0.001	No
12	0.37±0.40	0.98	0.989	<0.001	No
13	0.40±0.44	0.98	0.974	<0.001	No
14	0.46±0.49	0.98	0.979	<0.001	No
15	0.45±0.48	0.81	0.985	<0.001	No
16	0.40±0.46	0.82	0.992	<0.001	No
17	0.43±0.47	0.98	0.987	<0.001	No
18	0.43±0.46	0.97	0.985	<0.001	No
19	0.40±0.43	0.77	0.977	<0.001	No
20	0.39±0.43	0.80	0.972	<0.001	No
21	0.37±0.41	0.93	0.986	<0.001	No
22	0.47±0.49	0.94	0.991	<0.001	No
23	0.35±0.39	0.93	0.977	<0.001	No
24	0.44±0.47	0.96	0.987	<0.001	No
25	0.47±0.51	0.84	0.986	<0.001	No
25-Avg.	0.40±0.43[c]	0.94[d]	0.989	<0.001	No

a. Reference standard was a MetOne 063 thermistor within a 076B aspirated shield [2]. b. Shapiro-Wilk test for normality [3]. See the Materials and Methods Section for details. c. 25-average. d. Correlation r for HOBO #2 through #25 average vs. HOBO #1.

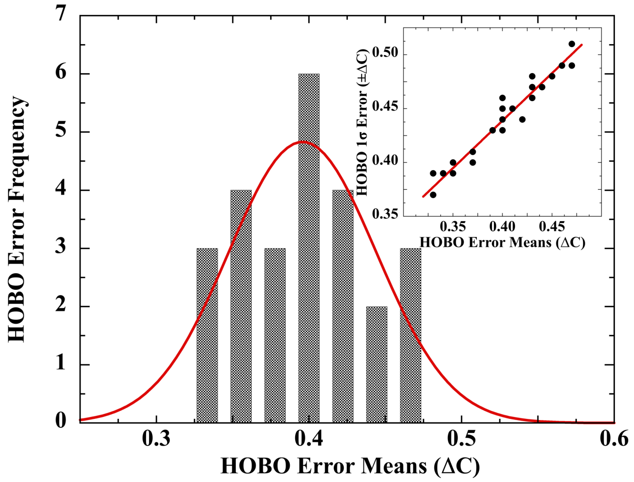

Figure S3: Histogram of HOBO error means. (red line), Gaussian fit to HOBO error means (μ = 0.396, $\sigma = \pm 0.048$, $r^2 = 0.69$). Inset: (points), HOBO error |σ| plotted against the error means. (red line), linear least squares fit: $y = 0.88\times(\text{error-mean})+0.087$, $r^2 = 0.95$.

The histogram profile is asymmetric and the Gaussian fit is a poor representation. The high correlation of the error means and error standard deviations (Figure S3, inset; r = 0.97) is inconsistent with the notions of random error or random error means.

Correlated means and standard deviations of systematic measurement error is suggestive that all twenty-five naturally ventilated HOBO sensors responded similarly to radiant heating of the sensor shield and wind speed effects. The aggregate measurement uncertainty, σ = ±0.40 C, is a valid uncertainty interval to apply to field meteorological air temperatures measured using a sensor within a naturally ventilated HOBO shield.

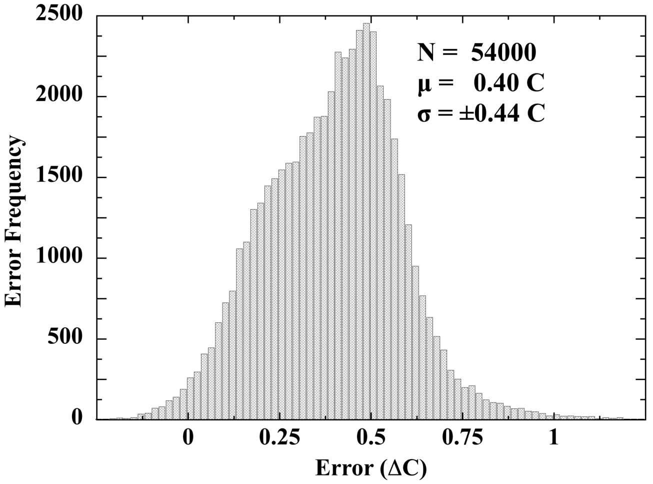

Figure S4. Histogram of the combined systematic measurement error from 25 naturally ventilated HOBO/thermistor air temperature sensors (N = 54000) [2].

III. Buoy-deployed Shield Calibration Study, Arabian Sea. The experiment consisted of sensors aboard a 3m diameter moored buoy deployed on the Arabian Sea. Temperature sensors were a vector averaging wind recorder (VAWR) which housed a thermistor in a 9-plate Gill radiation shield; a PRT in a naturally ventilated R. M. Young 12-plate shield (IMET AT), and two further thermistor air temperature sensors (IMET RH, and Standalone) in naturally ventilated shields [4, 5]. The thermistors were laboratory-calibrated to ±0.01 C. The reference sensor was a PRT in an R. M. Young aspirated shield, rated accurate to ±0.2 C in full sunlight. Measurements spanned October 1993-October 1994, off the coast of Oman. The data presented here derive from published Figure 3a. Measurement error is the differences of temperatures obtained from a naturally ventilated sensor and the aspirated R.M. Young PRT, (T_{NV} - T_{RMYasp}).

Figure S5 shows the digitized temperature measurement lines overlaid upon the published Figure 3a versions of IMET AT (digitized N = 111), IMET RH (digitized N = 101), STANDALONE (digitized N = 98) and VAWR (digitized N = 118) [4].

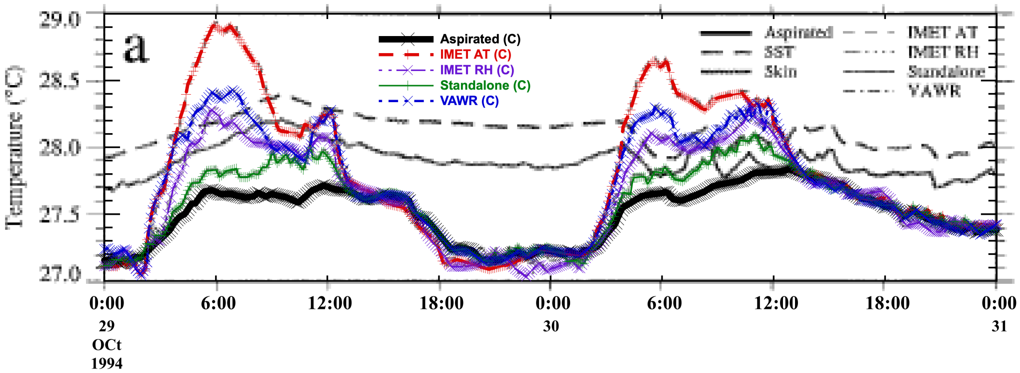

Figure S5: (colored points and colored lines), digitized reproduction of published Figure 3a IMET AT, IMET RH, STANDALONE and VAWR temperature measurements plotted to the same scale as, and overlaid upon, the original Figure 3a [4]. Colored text provides the identities. The published lines are present below the digitized points.

The overlay in Figure S5 illustrates the fidelity of the digitized points with the published temperature measurements. The independently digitized points were interpolated onto a uniform x-grid of 0.00 to 48.00, interval 0.1. The original data rate was, IMET AT, 1 min. average, N = 2880; IMET RH, 1 min. average, N = 2880; STANDALONE, 3.75 min. average, N = 768; AVWR, 3.75 min. average, N = 768. The digitized points (interpolated N = 481) under-sample the original data, but are uniformly dispersed, and faithfully reproduce the published phases and intensities.

In Figure S6 the air temperature measurement errors of the IMET AT, IMET RH and STANDALONE sensors are plotted against the measurement errors of the VAWR sensor.

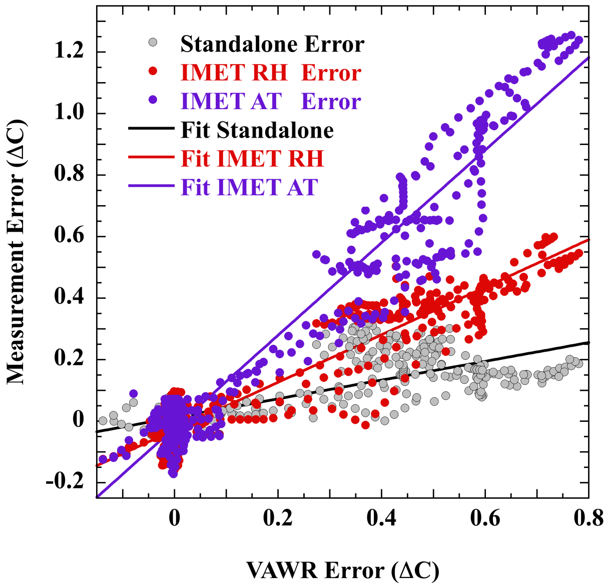

Figure S6: Correlation plot of measurement errors from four naturally ventilated sensors (see Figure S5) [4]. The identity of each sensor is shown on the face of the Figure. Each of three temperature measurement error data sets are plotted against the VAWR errors. An aspirated R. M. Young PRT sensor was reference. The lines are ordinary linear least square fits to the points. Standalone: $y = 0.305x+0.0115, r^2 = 0.63$; IMET RH, $y = 0.773x-0.0285, r^2 = 0.88$; IMET AT, $y = 1.505x-0.0220, r^2 = 0.94$.

The correlation of air temperature measurement errors among the four buoy-mounted naturally ventilated sensors is evident by inspection. Linear least square fits exhibit r-values indicating significant correlation, with mean VAWR correlation $r_{\square} = 0.90$. The Shapiro-Wilk test results are uniformly inconsistent with random error.

Table S9 provides the correlation matrix for the four naturally ventilated sensors and the results of the Shapiro-Wilk test for normality [4].

Table S9: Buoy Sensor Error Correlation Matrix and Shapiro-Wilk Test

Sensor	IMET AT	IMET RH	STANDALONE	VAWR	S-W(481)
IMET AT	1	0.943	0.766	0.969	0.834, p<0.001
IMET RH		1	0.860	0.936	0.887, p<0.001
STANDALONE			1	0.792	0.874, p<0.001
VAWR				1	0.823, p<0.001

The calibration standard was an Aspirated R. M. Young PRT sensor. Interpolated error N = 481; see Figure S5. Calibration r values were determined by pair-wise Student t-test. S-W is Shapiro-Wilk test for normality.

Figure S7 displays the distribution of error for each of the four sensors, each of which produced a diurnal distribution of errors.

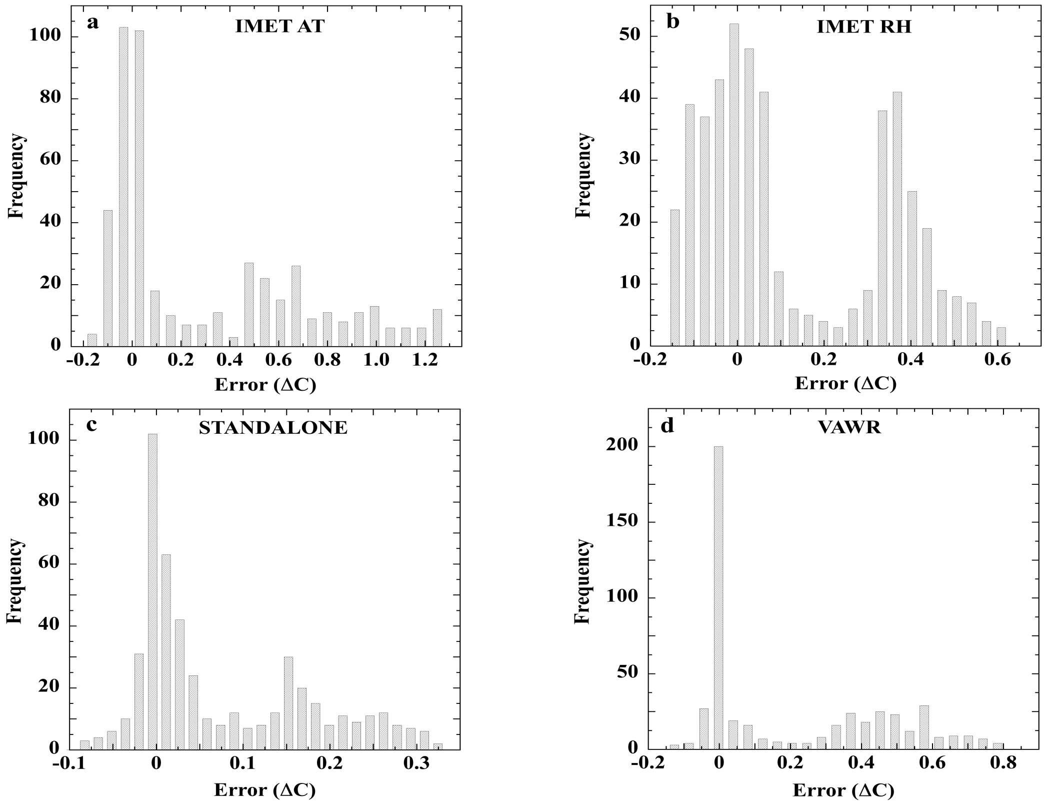

Figure S7: Histogram of air temperature measurement errors produced by each of four naturally ventilated sensors over a 2-day period aboard a 3-meter diameter floating buoy moored in the Arabian sea [4]. Points were digitized from Figure 3 (N = 481), thus the figures do not provide the original density of points (See Figure S5). [6]. Each error histogram is statistically non-normal.

To further the examination, the four sets of digitized measurement errors were appended into a single column file (N = 1924, $\mu = 0.17$ C, $\sigma = \pm 0.32$ C). These data are sufficient to coalesce into a normal distribution were the assumption of random error sustained. The histogram in Figure 8 shows that the combined set of sensor measurement errors retained the diurnal distribution of the four original error sets.

All day-time and all night-time errors were extracted from the combined data set by reference to the occurrence of inception and termination of short-wave radiation (*cf.* Figure 3b in [4]. The Shapiro-Wilk test results are provided in Table S10. Neither the day error nor the night error histogram was consistent with a normal distribution. Each error distribution was best-fit using a combination of Gaussian and Lorentzian lines (Table S10).

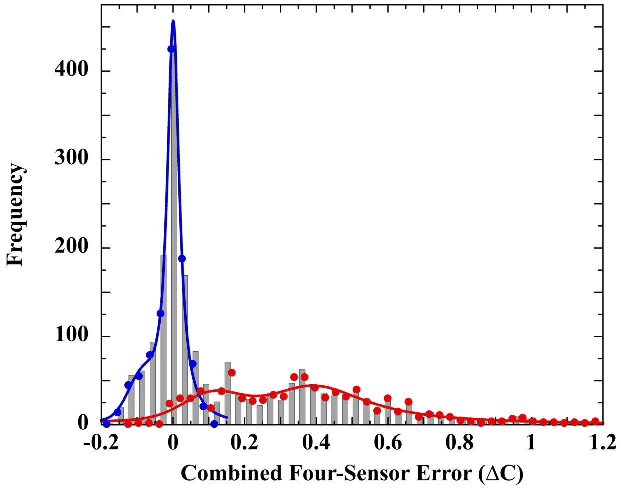

Figure S8: (Grey bars), histogram of the combined air temperature measurement error (N = 1924) of four sensors aboard a 3-meter buoy moored in the Arabian Sea off the coast of Oman. (Blue points), night-time error (N = 1024; S-W = 0.945, p<0.001); (red points) day-time error (N = 900; S-W = 0.945, p<0.001); (blue line), fit to night time error; (red line), fit to day-time error [6].

Table S10: Fitting Parameters for the Four-Sensor Measurement Error

Day Error; r^2 = 0.827		Night Error; r^2=0.997	
Gaussian	Lorentzian	Gaussian	Lorentzian
μ = 0.11	x_0 = 0.39	μ = -0.089	x_0 = 0.001
σ = ±0.078	Γ = 0.37	σ = ±0.038	Γ = 0.041
S-W(900) = 0.945, p < 0.001		S-W(1024) = 0.945, p = 0.003[b]	

The full data set and the day-time error are not normally distributed; a diagnosis clear merely by visual inspection. The night-time error is likewise not normally distributed, as diagnosed both by the Shapiro-Wilk test and by the fit. These results disconfirm the notion that air temperature measurements are subject only to random error.

IV. Stevenson screen on a sunny day, steady wind. Multiple screens, both commercial and custom, were tested on a clear sunny day at Ben Gurion University, Sde Boqer, Israel [7]. Wind was mild and fairly steady at 1-2 m/s. Both the Stevenson screen and an aspirated reference screen housed a Siemens M841 NTC thermistor, calibrated to ±0.05 C at 25 C [8].

Stevenson screen air temperature errors were digitized from published Figure 10 [7]. The 24 hours of air temperature sensor errors were compiled into a histogram of twenty-four 1-hour bins. Figure S9a displays the frequency histogram of error produced by the Stevenson screen over the 24-hour test period.

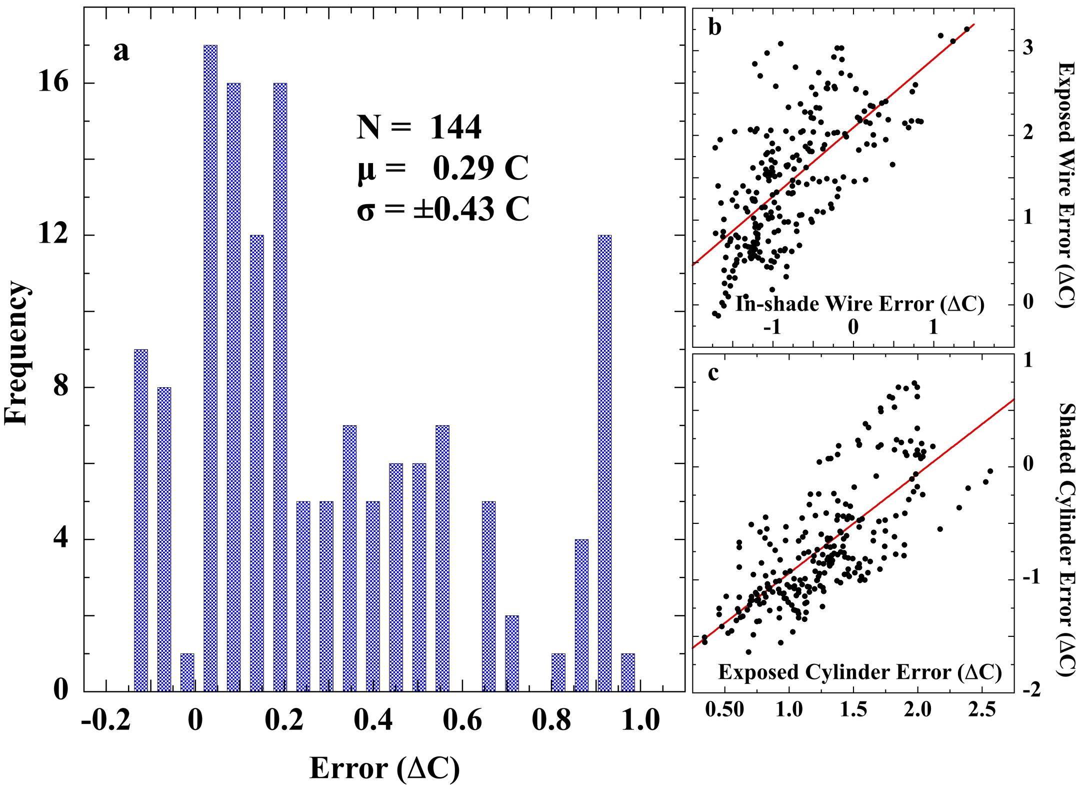

Figure S9: Panel a, Frequency histogram of air temperature measurement error of a calibrated Siemens M841 NTC thermistor housed within a naturally ventilated Stevenson screen [7]. Panel b, correlation plot (r = 0.67) of the systematic error produced by two bare copper-constantan wire thermocouple sensors. Panel c, correlation plot (r = 0.73) of the systematic error produced by two copper-constantan wire thermocouple sensors, each housed within a naturally ventilated custom cylindrical shield.

In all cases, the reference sensor was a Siemens M841 NTC thermistor housed in an aspirated custom shield [8]. The Stevenson screen errors yielded a Shapiro-Wilk test consistent with a non-normal distribution: S-W(145) = 0.913, errors $p < 0.001$.

Figure S9b is a correlation plot of the air temperature measurement errors produced by two bare copper-constantan thermocouple temperature sensors, one of which was housed beneath the roof of a shed (S-W(241) = 0.939, $p < 0.001$) while the other was exposed to full sun (S-W(241) = 0.982, p = 0.004). The red line is a linear least squares fit: $y = (0.81\pm0.06)x+2.09\pm0.06$; $r^2 = 0.45$.

Figure S9c is a correlation plot of two copper-constantan thermocouples, each housed in a naturally ventilated custom tubular shield. One instrument was shaded (S-W(243) = 0.935, $p < 0.001$) and the other was exposed to full sun (S-W(243) = 0.986, p = 0.020). The red line is a linear least squares fit: $y = (0.88\pm0.05)x-1.83\pm0.07$; $r^2 = 0.54$.

In no case are the results consistent with random measurement errors.

V. Calibration of a PRT within a MetSpec Double-Louver Modified Stevenson Screen. The experiment was carried out over 7 November 2019 to 20 August 2020 on a lawn surface at the Reading University Atmospheric Observatory [9]. The reference 1/3 Din PT100 Campbell Scientific PRT is specified to at least ±0.1 C accuracy. The measurement data set is available as Supporting Information provided by [9].

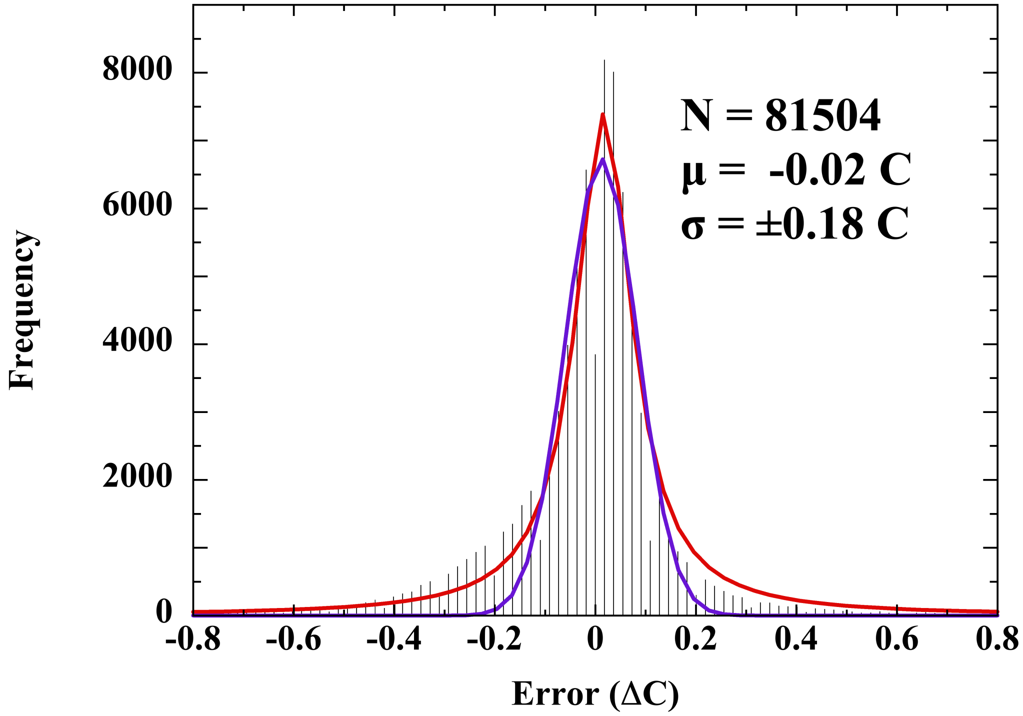

Figure S10: Histogram of air temperature measurement error produced by a PRT housed within a MetSpec "large" plastic Stevenson screen manufactured with double-louvers [9]. The error metrics appear on the face of the Figure. The full error range was -2.19 C to 1.75 C. (Red line), Lorentzian fit, $\Gamma = 0.136$ C, $\mu = 0.017$ C; fit $r^2 = 0.94$. (Violet Line), Gaussian fit, $\sigma = \pm 0.071$ C, $\mu = 0.124$ C; fit $r^2 = 0.92$.

The very large measurement error data set (N = 81504) is sufficient to have produced a normal distribution, did it consist of random error. Neither a Lorentzian nor a Gaussian line provided a good fit to the distribution. However, from visual inspection alone, the Gaussian fit is clearly much poorer in the wings.

VI. Bucket minus Bucket distributions. Figure S11 compares Gaussian and Lorentzian fits to inter-bucket SST differences in consecutively measured samples of marine waters [10]. The meteorological buckets were manufactured of wood, or canvas, or rubber (see text for details).

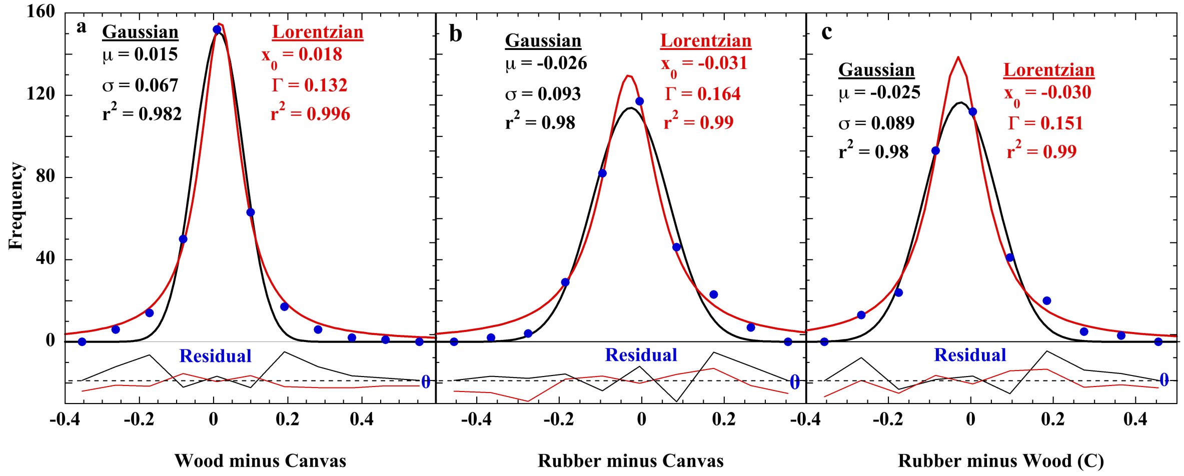

Figure S11: Comparison of Gaussian or Lorentzian fits to histograms of the differenced bucket SST measurements [10]. In panel a, the Lorentzian alone provided a reasonable fit, although not as well as the mixed Lorentzian-Gaussian fit (compare with Figure 12). In panels b and c, a Gaussian or Lorentzian fit alone yielded equivalent goodness-of-fit r^2 statistics. However, neither fit fully reproduced the wings of the distribution. The foot of each panel shows the color-coded fit residuals. The residual zero is the horizontal dashed line.

In all cases, the fit with a combination of Gaussian and Lorentzian lines more closely matched the asymmetry of the histogram than a fit using a single Lorentzian or Gaussian. See the text for the results of Shapiro-Wilk normality tests of these data.

VII. WMO Bucket and Engine Intake Temperature Survey. Comparison of Gaussian and Lorentzian fits to the engine-intake minus bucket SST differences obtained from the World Meteorological Organization (WMO) global survey [11]. See text for details.

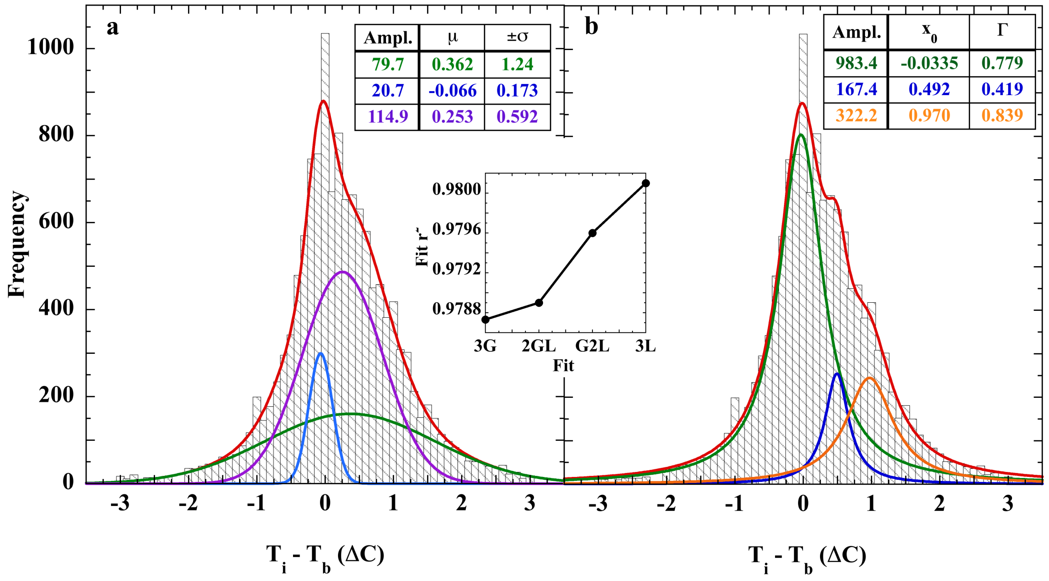

Figure S12: Histogram of arithmetic difference of SSTs (N = 13511) measured during the WMO global survey [11]. Temperatures were measured either at the ship engine cooling water intake (T_i) or from a meteorological bucket sample (T_b). Panel a: best fit using three Gaussians. Panel b, best fit using three Lorentzians. The fitted parameters are given on the face of each panel. Inset: improvement in the fit r^2 as Gaussians are step-wise replaced with Lorentzians. Although the improvement is minor, the positive trend is apparent. "Ampl." is amplitude.

VIII Walden 1966, 25-49.9 °N, S (T_b-T_E). Frequencies of bucket minus engine intake SST differences were expressed in latitudinal bands and separated according to 0-1 Bft, 2-4 Bft, 5-7 Bft and >8 Bft wind strength, where Bft is Beaufort (0.836 ms^{-1}) [12]. For this work, the wind-strength SST differences were digitized from the Walden Figure 1. For the presentation here, the four sets of digitized (T_b-T_E) differences were interpolated onto a common ΔT x-grid. The interpolated (T_b-T_E) values were summed to produce the frequencies representing all wind-speeds.

Figure S13 shows the combined wind-strength frequencies of differences representing the frequencies of (T_b-T_E) for 25-49.9° North and South latitude and all wind strengths.

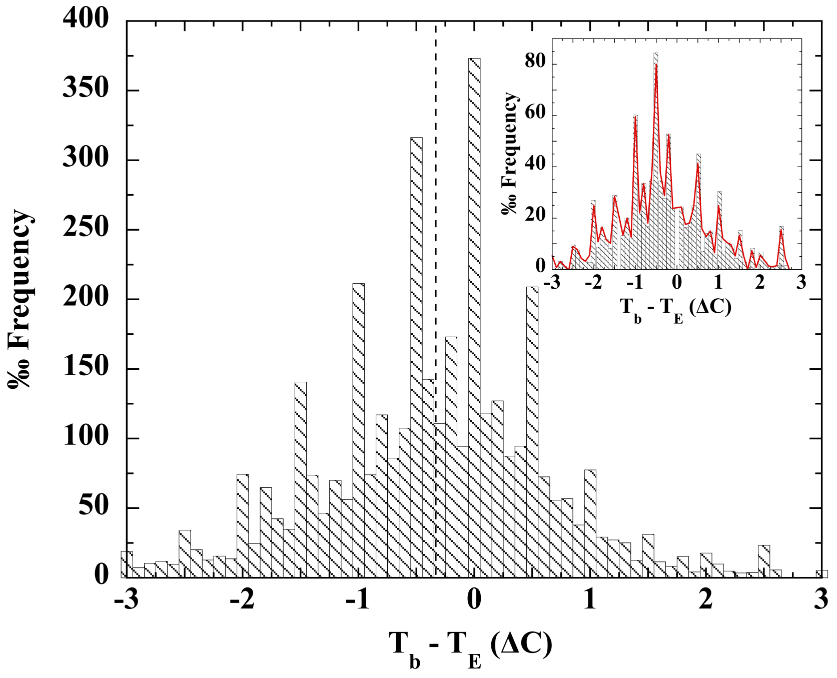

Figure S13: Frequency of (T_b-T_E) for 25-49.9° North and South latitude at all wind-speeds, digitized and combined from Walden Figure 1 [12]. Outliers beyond ±3.0 C are excluded. The dashed line marks the arithmetic mean. Inset: (bars), the 0-1 Bft (T_b-T_E), as digitized; (red line), the same 0-1 Bft differences after interpolation onto the common x-grid. The overlay illustrates the fidelity of the interpolated data to the data digitized from the published figure.

The 25-49.9° North and South latitude frequencies of (T_b-T_E) over ±3.0 C yielded an arithmetic mean μ = -0.34 C and standard deviation σ = ±1.0 C.

These differenced errors should produce a normal distribution were the underlying error data sets normally distributed. The lack of normality results again disconfirm the notion that SSTs include only random measurement error.

IX. References

1. Brandsma, T., and J. P. van der Meulen. "Thermometer Screen Intercomparison in De Bilt (the Netherlands) Part Ii: Description and Modeling of Mean Temperature Differences and Extremes." *Int. J. Climatol.* 28, no. 3 (2008): 389-400.
2. Mauder, Matthias, R. L. Desjardins, Zhiling Gao, and Ronald van Haarlem. "Errors of Naturally Ventilated Air Temperature Measurements in a Spatial Observation Network." *Journal of Atmospheric and Oceanic Technology* 25, no. 11 (2008): 2145-51.
3. Shapiro-Wilk. "Shapiro-Wilk Test Calculator." Statistics Kingdom, https://www.statskingdom.com/shapiro-wilk-test-calculator.html (accessed 27 February).
4. Anderson, Steven P., and Mark F. Baumgartner. "Radiative Heating Errors in Naturally Ventilated Air Temperature Measurements Made from Buoys." *Journal of Atmospheric and Oceanic Technology* 15, no. 1 (1998): 157-73.
5. Hosom, David S., Robert A. Weller, Richard E. Payne, and Kenneth E. Prada. "The Imet (Improved Meteorology) Ship and Buoy Systems." *Journal of Atmospheric and Oceanic Technology* 12, no. 3 (1995): 527-40.
6. Razali, Nornadiah Mohd, and Yap Bee Wah. "Power Comparisons of Shapiro-Wilk, Kolmogorov-Smirnov, Lilliefors and Anderson-Darling Tests." *Journal of statistical modeling and analytics* 2, no. 1 (2011): 21-33.
7. Erell, Evyatar, V√≠tor Leal, and Eduardo Maldonado. "Measurement of Air Temperature in the Presence of a Large Radiant Flux: An Assessment of Passively Ventilated Thermometer Screens." *Boundary-Layer Meteorology* 114, no. 1 (2005): 205-31.
8. Erell, Evyatar. 12 March 2023.
9. Harrison, R. Giles, and Stephen D. Burt. "Quantifying Uncertainties in Climate Data: Measurement Limitations of Naturally Ventilated Thermometer Screens." *Environmental Research Communications* 3, no. 6 (2021): 061005.
10. Matthews, J. B. R., and J. B. Matthews. "Comparing Historical and Modern Methods of Sea Surface Temperature Measurement – Part 2: Field Comparison in the Central Tropical Pacific." *Ocean Sci.* 9, no. 4 (2013): 695-711.
11. James, Richard W., and Paul T. Fox. "Comparative Sea-Surface Temperature Measurements (Wmo-No. 336): Results of a Programme of Comparative Measurements Conducted under the Auspices of the Commission for Marine Meteorology." In *Reports on Marine Science Affairs*, edited by Paul T. Fox, ix, 27. Geneva: Commission for Marine Meteorology, World Meteorological Organization 1972.
12. Walden, Hans. "On the Measurement of Water Temperature on Merchant Vessels." *Deutsche Hydrografische Zeitschrift* 19, no. 1 (1966): 21-28.
